# A Study of Publicly Available Resources Addressing Legal Data-Sharing Barriers: Systematic Assessment

**DOI:** 10.2196/39333

**Published:** 2022-09-06

**Authors:** Mohammad A Karim, Hye-Chung Kum, Cason D Schmit

**Affiliations:** 1 Department of Health Policy and Management School of Public Health Texas A&M University College Station, TX United States; 2 Population Informatics Lab School of Public Health Texas A&M University College Station, TX United States; 3 Health Law and Policy Program School of Public Health Texas A&M University College Station, TX United States

**Keywords:** privacy, confidentiality, public health informatics, data sharing

## Abstract

**Background:**

United States data protection laws vary depending on the data type and its context. Data projects involving social determinants of health often concern different data protection laws, making them difficult to navigate.

**Objective:**

We systematically aggregated and assessed useful online resources to help navigate the data-sharing landscape.

**Methods:**

We included publicly available resources that discussed legal data-sharing issues with some health relevance and published between 2010 and 2019. We conducted an iterative search with a common string pattern using a general-purpose search engine that targeted 24 different sectors identified by Data Across Sectors for Health. We scored each online resource for its depth of legal and data-sharing discussions and value for addressing legal barriers.

**Results:**

Out of 3710 total search hits, 2721 unique URLs were reviewed for scope, 322 received full-text review, and 154 were selected for final coding. Legal agreements, consent, and agency guidance were the most widely covered legal topics, with HIPAA (The Health Insurance Portability and Accountability Act), Family Educational Rights and Privacy Act (FERPA), Title 42 of the Code of Federal Regulations Part 2 being the top 3 federal laws discussed. Clinical health care was the most prominent sector with a mention in 73 resources.

**Conclusions:**

This is the first systematic study of publicly available resources on legal data-sharing issues. We found existing gaps where resources covering certain laws or applications may be needed. The volume of resources we found is an indicator that real and perceived legal issues are a substantial barrier to efforts in leveraging data from different sectors to promote health.

## Introduction

Increasingly, data are leveraged to promote health outcomes, and practitioners are increasingly using data from different sectors to address social determinants of health. Unfortunately, the United States does not have a comprehensive data protection law; instead, there is a patchwork of laws that vary depending on the data type, who has it, and what they want to do with it [1-3]. Consequently, federal data protection laws vary considerably, and these differences are magnified by differences between state and local governments within the United States. The variation in data protection laws is particularly vexing for efforts in promoting data sharing to promote population health [4]. For example, the absence of a public health exception in the Title 45 of the Code of Federal Regulations (CFR) Part 2 protections for substance abuse treatment data has posed a major challenge to leveraging data to combat the opioid epidemic [5]. Commonly, practitioners are confronted with legal barriers to data use; some are real barriers (eg, legal language prohibiting data use), but many are perceived legal barriers (eg, perceptions that laws restrict data use) [2,6]. For example, the HIPAA (Health Insurance Portability and Accountability Act) rule has a robust public health data use exception [7], but it has been frequently cited as a (perceived) barrier to sharing data for public health purposes (eg, the 2013 fungal meningitis outbreak) [6]. Practitioners pursuing multisectoral data projects are forced to navigate the real and perceived legal barriers from the patchwork of US data protection laws [1,2,8].

Public health practitioners are specially affected because a combination of data on different aspects of a person’s life and health may prove necessary to make the best-informed decisions. For example, education is a potent social determinant of health [9]. Consequently, there is substantial interest in determining whether laws (eg, the Family Educational Rights and Privacy Act [FERPA] and HIPAA) permit linking education attainment data with health outcomes data to further understand this social determinant of health [10-12]. Importantly, while leveraging data for public health has support among the US public, many data silos are often reinforced by legal restrictions. This sometimes leads to suboptimal cross-sector collaboration, ultimately resulting in less-than-ideal population health efforts. Nevertheless, navigating these different legal data protection frameworks is essential to achieving the goal of implementing precision public health because data on social determinants of health (eg, education, crime, and housing) will implicate several different data protection laws [8].

For example, previous studies have indicated the value of data linkage in identifying the association between health and health determining factors, such as income and crime [13,14]. Among recent initiatives, the efforts to link National Center for Health Statistics (NCHS) data and US Department of Housing and Urban Development (HUD) administrative records is an example of collaboration between 2 federal agencies that enabled linkage of housing and health data where the agencies used a memorandum of understanding to comply with relevant regulations [15]. Detailed guidance on addressing the legal challenges involved in these types of data sharing and linking efforts can provide useful reference points for practitioners at the state and local levels.

Publicly available online resources can help practitioners navigate these issues and inform conversations with legal counsel. Publicly available resources can help practitioners to understand whether laws exist that might protect certain data (eg, education, substance abuse treatment, juvenile justice, and government nutrition program data) [16]. However, without more detailed discussion, general descriptions of laws could beget perceived data-sharing barriers that could discourage pursuit of a proposed data-sharing project. However, laws that protect data often permit data to be used for secondary purposes [8,17]. Consequently, the most valuable and helpful publicly available resources on legal data-sharing issues contain detailed discussion of data protection laws, including both restrictions and permissions [18]. Detailed publicly available resources are becoming increasingly important as health informatics projects begin to span data sources in the effort to understand the social determinants of health.

In the field, publicly available resources are often the first resort (ie, Google searches). The presence or absence of quality resources describing legal mechanisms for data sharing can impact decisions to pursue data-sharing projects for public health purposes. Ideally, a resource goes beyond identifying legal issues and actually applies the law to specific use cases [18]. This type of use-case analysis can help public health professionals understand what is legally possible and help professionals identify relevant legal issues to discuss with their legal counsel. Although following professional legal advice is imperative for any data-sharing project, the existence of publicly available legal resources can be highly influential in the earliest planning stages and can sway leadership decisions on whether to pursue official legal counsel or abandon a project idea at inception.

However, finding quality resources discussing legal data-sharing issues can be challenging. For example, many documents discussing data sharing may make a passing reference to challenges posed by privacy laws and may even name a law (eg, HIPAA) [19-21]. However, quality (and helpful) legal analysis usually requires applying laws to facts using case studies or examples to show how the law operates in given situations [22,23]. Documents that only superficially reference privacy or legal data-sharing barriers are not helpful to practitioners and may even bury quality resources in search results.

There have been efforts by different organizations to facilitate data sharing across sectors, and many approaches have been documented. For example, in 2017, Data Across Sectors for Health (DASH) and the Network for Public Health Law developed the Legal Bibliography and more recently the DASH Knowledge Base, an online database of publicly available data-sharing resources to help public health practitioners navigate these complex legal issues [24]. This review is an extension of this work. However, these resources have not been systematically studied. Understanding this landscape is critical to understanding practitioners’ current focus areas, specific challenges and needs, and what gaps exist in the existing literature.

This review focuses entirely on public resources (eg, white papers, reports, toolkits, and open-access academic articles) that are freely available to laypersons and practitioners. Prior reviews have explored data-sharing issues, but these are mostly academically focused (ie, sharing research data between academics) [25]. Reviews of resources concerned with combating legal barriers of data sharing in nonresearch settings are nonexistent in academic literature.

In this review, we have aggregated and screened through those publicly available resources that may help public health officials and practitioners navigate the data-sharing landscape. In recognition that health is affected by a tremendous number of factors (eg, social determinants of health) and assuming that future public health informatics application (eg, precision public health) can leverage these data for public health purposes, we were inclusive to the broadest extent in identifying resources that cover sectors affecting an individual’s health.

## Methods

### Scope

We collected publicly available internet resources discussing data-sharing legal issues relevant to health. We used a broad interpretation of factors affecting health, considering any factor directly or indirectly affecting the well-being of an individual as a potential determinant of health. We only included resources if they were free to access and publicly available (including open-access academic articles). Academic articles were only included if they met our inclusion criteria and were freely accessible. We omitted results published prior to 2010 to ensure that the resources were reasonably current; laws change, and at least one major health-related data-sharing law, the Health Information Technology for Economic and Clinical Health Act, was enacted in 2009. We also excluded resources where the law or legal issue was not discussed with particularity; that is, resources that merely referenced a law or legal issue without some discussion were omitted. Some documents—like news articles, unannotated legislative text, and organizational policy statements—were excluded because they were not developed as “resources.”

### Collection

We used a general-purpose search engine (Google) to identify the resources because a consumer-focused search engine is likely to be a common (if not default) search tool used by practitioners to learn about data-sharing issues.

To ensure a comprehensive search scope, we developed a complex search pattern yielding 75 individual searches, rather than using a single search term. Each search included a common string pattern: (common search stem) + (sector) + (data protection term). The common search stem applicable to each search was as follows: (“data sharing” OR “data use” OR “information sharing” OR “information use”) + (“law” OR “regulation” OR “legal” OR “statute”). We identified search terms to target a total of 24 different sectors (sectors were identified in collaboration with the Data Across Sectors for Health and the Network for Public Health Law) [26], and an additional set of searches was executed without a specified sector (24 sectors and 1 overall). The common search stem and the sector search terms were executed a total of 3 times, each with a different data protection term: “privacy,” “confidentiality,” or “consent” (in that order). This search pattern yielded a total of 75 individual searches (ie, 25×3=75), and the first 50 hits for each search were saved. We justified capping our individual search results at 50 on the basis that individuals do not often view more than 5 pages of Google search results. The initial search was completed in September 2019.

### Coding

Two researchers (CS and MK) coded each resource independently. One researcher (CS) had a legal background and expertise in legal data-sharing issues, and the other researcher (MK) had a health services research background with expertise in data analysis. We used coding meetings to resolve discrepancies.

We scored each online resource on a scale of 1 to 4 (lowest 1, highest 4) in terms of their depth of legal issues discussed, depth of data-sharing discussion, and value for addressing legal barriers. We calculated interrater reliability scores for these 3 measures using Gwet’s AC2 for ordinal data [27]. We used the objective benchmarking standards proposed by Altman [27] to interpret the AC2 coefficients (where a score of <0.20 represented “Poor”, 0.21 to 0.40 represented “Fair”, 0.41 to 0.60 represented “Moderate”, 0.61 to 0.80 represented “Good”, and 0.81 to 1.00 represented a “Very Good” strength of agreement) [27]. The calculated Gwet’s AC2 scores for depth of legal discussion and overall value of resource were 0.59, indicating that agreement on these 2 measures approached the “Good” strength of agreement benchmark. The AC2 score for depth of data-sharing discussion was lower at 0.40, indicating that agreement on this measure approached the “Moderate” strength of agreement benchmark. The following section contains brief descriptions of our coding criteria for these 3 items. However, Multimedia Appendix 1 describes the coding criteria in greater detail.

Codes for the depth of legal discussion were primarily determined by the presence and extent of 2 factors: (1) discussion or description of the law or legal issues and (2) application of the law or legal issue on a specific set of facts (eg, à la tradition legal analysis). For example, a resource that contained both a detailed description of the law and applied the law to a specific use case would earn the highest score of 4 for legal depth. However, if a resource either described the law in detail or provided an extended discussion of how the law was applied in specific use cases, but did not do both, the score the resource received was lowered to 3 instead of 4. In contrast, a resource that identified the law or legal issue related to specific use cases and provided only basic information about the law earned a score of 2, whereas a resource that contained only a superficial description of the law or legal issue earned the lowest score of 1. In addition to these criteria, we had another criterion for template legal agreements. Template agreements with extensive annotations (ie, explaining the purpose or function of contractual terms) were coded with the highest legal depth (score of 4), and template agreements with moderate or without annotations were scored lower (score of 3 or 2, respectively). Importantly, the coding of the depth of legal discussion did not consider the quality or legal accuracy of the discussion nor whether the discussion appears consistent with the referenced statutes, regulations, or related judicial interpretations.

The coding on the depth of data-sharing discussion evaluated the extent the resource covers strategies to initiate or maintain at least 1 type of data-sharing activity. Codes for the depth of data-sharing discussion were primarily determined by the presence and extent of 2 factors: (1) discussion or description of a data-sharing issue and (2) discussion or description of a data-sharing strategy or process. Two additional factors separated the highest-scoring resources on data sharing: (1) use cases explaining data-sharing issues and strategies in specific contexts and (2) links to recommended additional data-sharing resources.

Finally, we assessed the value of each resource for addressing legal barriers to nonexpert users. The codes on the overall value of the resource for addressing legal barriers were based on the presence of several factors. Some factors weighed in favor of higher scores, including if the resource was highly scored for legal or data-sharing discussion, user-friendly, or from an official governmental source. Other factors weighed against a higher score, including if the resource contained only limited context (eg, PowerPoint slides) or if the relevant discussion was only tangential to the focus of the resource (eg, a resource that includes an overview of legal or data-sharing issues as an appendix to the main document).

## Results

### Overview

**Figure 1:**
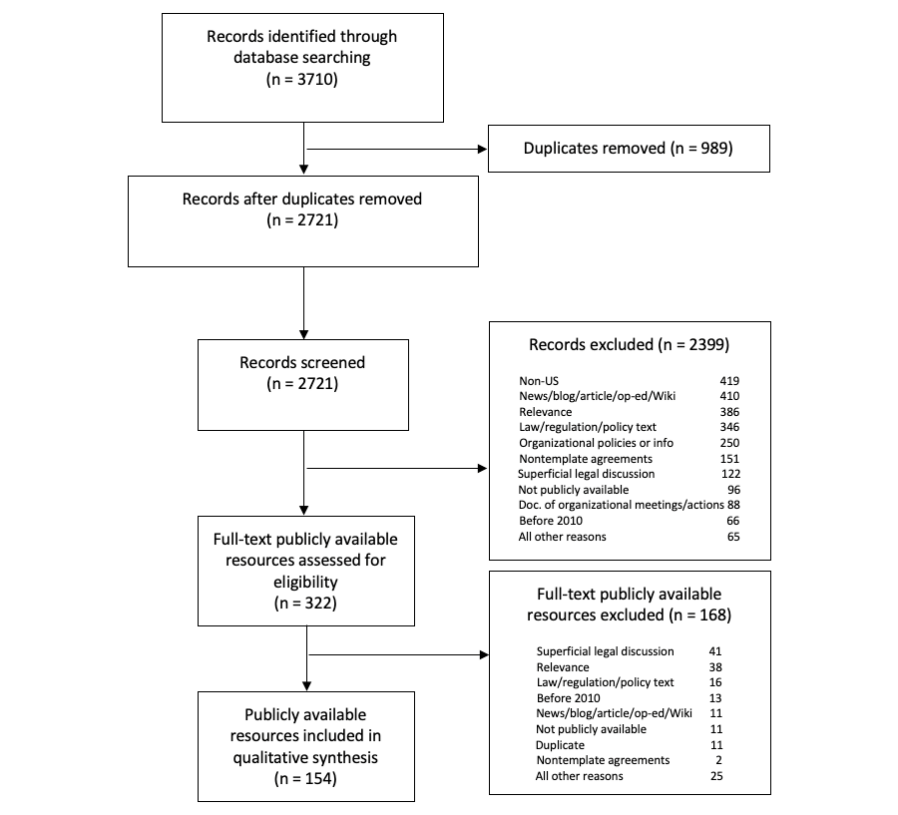
PRISMA (Preferred Reporting Items for Systematic Reviews and Meta-Analyses) chart showing the scoping process for collected records and publicly available resources. doc: document; Wiki: Wikipedia.

Our sector-specific searches provided a total of 3710 hits, out of which 989 were duplicates. After removing the duplicates, the remaining 2721 unique URLs were subjected to scoping screening. The full text of 322 in-scope resources were reviewed, out of which 154 were selected for final coding (Figure 1). Table S1 in Multimedia Appendix 2 includes a list of all included resources, their sectors covered, and their scores for legal depth, data-sharing depth, and value.

**Figure 2:**
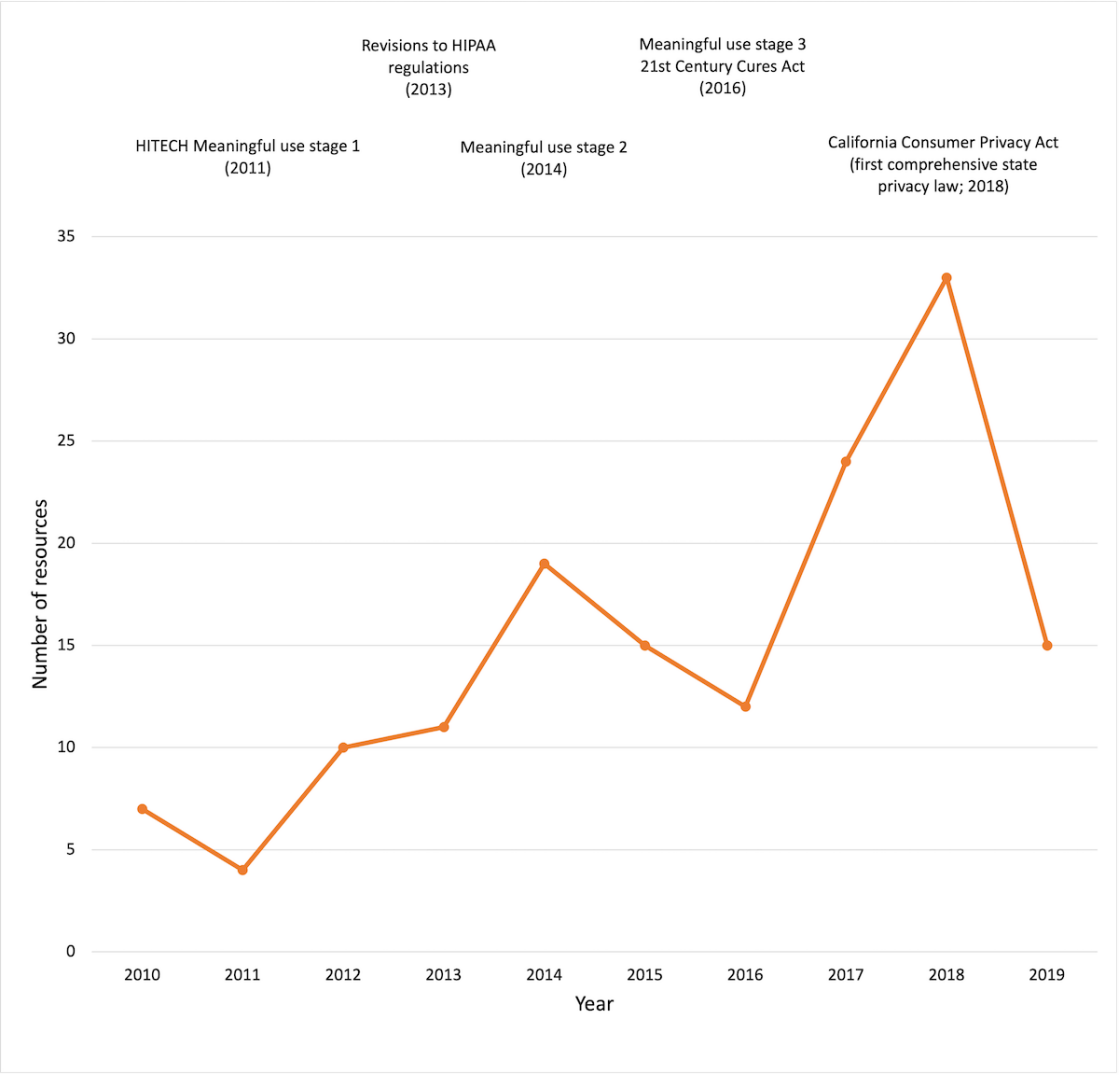
Number of publicly available resources identified per year and significant legal developments in privacy. Note that the 2019 data represent a partial year (January to September) with average monthly resources dropping from 2.75 in 2018 to 1.67 in 2019. Four resources identified in our 2019 search were subsequently updated in 2020 prior to coding completion. HITECH: Health Information Technology for Economic and Clinical Health Act. HIPAA: The Health Insurance Portability and Accountability Act.

Common resources excluded were company privacy statements, commentaries or analysis [28], slideshow documents with superficial information [29], and sources that have no discussion of US law [30,31]. Among the resources selected for coding, an upward trend in number between the years 2010 and 2018 was observed (Figure 2).

### Legal Topics and Resources Covered in Resources

Legal agreements for data sharing (n=63) were the most commonly available resource, followed by consent (n=57) and agency guidance (n=57; Table 1). Among those legal topics covered in at least 10 resources, legal case studies had the highest mean scores in terms of legal depth (mean score 3.38, SD 0.75), data-sharing depth (mean score 3.12, SD 1.11), and resource value (mean score 3.31, SD 0.79).

**Table 1:** Legal subject or topic covered by publicly available resources, mean depth of legal discussion and data-sharing discussion, and mean resource value (N= 154).

Legal resource	Resources present in, n	Legal depth score, mean (SD)	Data-sharing depth score, mean (SD)	Resource value score, mean (SD)
Legal agreements for data sharing	63	2.90 (0.91)	2.57 (1.10)	2.94 (0.86)
Consent (obtaining consent, waiving requirements, models)	57	3.12 (0.85)	2.68 (1.07)	3.05 (0.89)
Agency resource or guidance	57	3.00 (0.85)	2.12 (1.13)	2.81 (0.93)
Interagency data sharing	50	3.14 (0.90)	2.86 (1.09)	3.00 (0.97)
General legal overview (ie, no specific application indicated)	41	2.88 (0.93)	1.98 (1.06)	2.51 (0.98)
Resource links	34	3.09 (0.83)	2.79 (1.12)	3.06 (0.81)
Case studies applying law	26	3.38 (0.75)	3.12 (1.11)	3.31 (0.79)
Provider sharing	22	3.00 (0.87)	2.82 (1.01)	2.91 (1.02)
Health authority use	22	3.18 (0.85)	2.82 (0.96)	3.09 (1.06)
Health information exchange	19	3.00 (0.94)	2.63 (1.07)	2.84 (1.01)
Frequently asked questions about law	14	3.21 (0.70)	2.71 (1.27)	2.93 (1.00)
Other legal resource	13	2.92 (1.04)	2.69 (1.18)	2.85 (1.34)
Data system governance	8	2.75 (0.71)	3.50 (0.53)	3.00 (0.93)
Data sharing for program evaluation	7	3.57 (0.53)	3.57 (0.79)	3.71 (0.49)
Court orders/subpoenas	4	3.25 (0.50)	3.25 (0.96)	3.50 (0.58)
Working with legal counsel	4	2.25 (0.96)	3.25 (0.96)	3.00 (0.82)
Medical-legal partnerships	2	3.50 (0.71)	3.50 (0.71)	3.50 (0.71)
Model legislation	2	2.00 (0.00)	1.50 (0.71)	1.50 (0.71)
Statistical methods for protecting privacy and confidentiality	1	2 (N/A^a^)	4 (N/A)	3 (N/A)

^a^N/A: not applicable.

### Laws Discussed in Resources

The reviewed resources covered a total of 96 laws or legal issues to different degrees, but the plurality of them focused on only a handful of laws. Out of the 97 laws discussed in total, only 16 were discussed in at least 4 resources (Table 2). HIPAA (n=74), FERPA (n=41), and 42 CFR Part 2 (n=35) were the top 3 federal laws discussed in the resources [32,33]. See Table S2 Multimedia Appendix 2 for laws discussed in fewer than 4 resources. Among the laws discussed in at least 10 resources, the Privacy Act of 1974 scored the highest mean scores in terms of legal depth (mean score 3.45, SD 0.69), and FERPA scored the highest on data-sharing depth (mean score 2.95, SD 1.09) and resource value (mean score 3.22, SD 0.88). Among the 154 full-text resources coded, 68.8% (n=106) discussed more than 1 law (Supplemental Figure S1, Multimedia Appendix 2).

**Table 2:** Laws discussed in publicly available resources, mean depth of legal discussion and data-sharing discussion, and mean resource value (N=154).

Law	Resources present in, n	Legal depth score, mean (SD)	Data-sharing depth score, mean (SD)	Resource value score, mean (SD)
Health Insurance Portability and Accountability Act	74	3.01 (0.88)	2.65 (1.13)	2.93 (0.94)
Other state or local law(s)	43	2.91 (0.92)	2.37 (1.09)	2.79 (0.94)
Family Educational Rights and Privacy Act	41	3.24 (0.83)	2.95 (1.09)	3.22 (0.88)
42 CFR^a^ Part 2	35	3.26 (0.82)	2.91 (1.07)	3.09 (0.95)
General legal concepts	22	2.27 (1.03)	2.18 (1.10)	2.45 (1.06)
The Privacy Act of 1974	11	3.45 (0.69)	2.64 (1.43)	3.18 (0.98)
Health Information Technology for Economic and Clinical Health Act	10	3.10 (0.99)	2.80 (1.03)	2.80 (0.92)
Freedom of Information Act (or similar state laws)	9	3.44 (0.53)	2.56 (1.33)	3.00 (1.12)
Medicaid privacy requirements	8	2.62 (0.92)	2.38 (0.92)	2.75 (1.04)
Federal Policy for the Protection of Human Subjects (Common Rule)	8	2.38 (0.92)	2.12 (1.13)	2.25 (0.89)
Workforce Innovation and Opportunity Act	7	3.71 (0.49)	2.71 (1.11)	3.43 (0.79)
Confidentiality protections governing unemployment compensation wage records	5	3.40 (0.55)	2.80 (1.30)	3.40 (0.89)
The McKinney-Vento Homeless Assistance Act of 1987	5	2.60 (0.89)	2.80 (1.10)	2.80 (0.84)
Individuals with Disabilities Education Improvement Act	4	3.25 (0.96)	3.25 (0.96)	3.25 (0.96)
Confidential Information Protection and Statistical Efficiency Act of 2002	4	3.50 (0.58)	3.25 (0.96)	3.50 (0.58)
Food Stamp Act of 1964	4	2.75 (0.50)	2.50 (1.00)	2.75 (0.50)

^a^CFR: Code of Federal Regulations.

### Data Use Cases Covered in Resources

The most frequently addressed use case was record matching across systems with 113 resources discussing this (Table 3). Statistical analysis was the second most discussed use case (n=58). This was followed by reporting function, a use case that was discussed in 44 resources. Among the data use cases referenced in at least 10 resources, calculating and reporting metrics scored the highest for legal depth (mean score 3.40, SD 0.71) and resource value (mean score 3.36, SD 0.70), while generating predictive scores scored the highest on data-sharing depth (mean score 3.00, SD 1.20).

**Table 3:** Data use case discussed in publicly available resource, mean depth of legal discussion and data-sharing discussion, and mean resource value (N=154).

Use case	Resources present in, n	Legal depth score, mean (SD)	Data-sharing depth score, mean (SD)	Resource value score, mean (SD)
Using identifying information to match records across systems to create a more encompassing view of a person or case	113	2.90 (0.97)	2.62 (1.12)	2.85 (0.99)
Statistical analysis to look for useful patterns and relationships in the data set	58	3.12 (0.86)	2.79 (1.10)	3.09 (0.88)
Reporting functions that allow users to specify and generate reports using items from a menu	44	3.11 (0.84)	2.45 (1.21)	3.05 (0.86)
Calculating and reporting of metrics, indicators, and dashboards enabling group comparison and tracking of progress over time	25	3.40 (0.71)	2.68 (1.31)	3.36 (0.70)
Generating scores that predict/identify likelihood or risk of future events	22	3.14 (0.77)	3.00 (1.20)	3.23 (0.87)
Automating decision support and generating recommendations or alerts	21	3.14 (0.91)	2.38 (1.28)	2.95 (0.92)
Not expressly discussed in resource	14	2.64 (0.93)	1.79 (0.97)	2.50 (0.94)
Presentation and visualization of data such that the viewer grasps the relevance of the information	10	2.70 (0.82)	2.00 (1.25)	2.60 (0.97)
Other use case	8	2.88 (0.99)	1.88 (1.36)	2.88 (0.83)
Mapping/geographic information systems—analysis of data by geographic location and presentation as maps	4	3.25 (0.96)	2.75 (1.50)	3.50 (1.00)

### Sectors Covered in Resources

A total of 20 sectors were covered as the primary focus of the included resources, among which education (n=22), public health (n=16), and academia (n=13) were most common. Among these 3, education was the highest-scoring sector in terms of legal depth (mean score 3.09, SD 0.92), data-sharing depth (mean score 2.82, SD 1.22), and resource value (mean score 3.05, SD 0.95). The 4 sectors that were initially searched but not considered as a representative sector for any of the resources in the final data set were “elected or appointed official,” “faith or faith based,” “parks and recreation,” and “philanthropy.” Among the sectors, clinical health care was the most prominent sector with a mention in 73 websites or files (Table 4). Among the 154 resources, around 86.4% (n=133) discussed more than 1 sector (Figure S1, Multimedia Appendix 2).

A relatively small proportion of our 2721 unique search results were scored as having the greatest depth of legal discussion (n=48, 1.76% among unique search results) [32,33] and depth of data-sharing discussion (n=36, 1.32% among unique search result). Instead, most search results were either international (eg, did not address US laws) [30], out-of-date, provided only legislative updates [34], had a specific focus unrelated to health, or only contained passing or superficial discussion of legal data-sharing issues. Additionally, we found resources that were blog posts [35], PowerPoint slides with very limited information, privacy statements on commercial sites [36], policy memoranda [37], or organization-specific policies [38], or that only defined a law without providing any further discussion [39].

**Table 4:** Sectors addressed as the main or primary focus of a resource, number of resources addressing the sector as a secondary focus, mean depth of legal discussion and data-sharing discussion, and mean resource value (N=154).

Sector	Primary focus					Secondary focus
	Resources present in, n	Legal depth score, mean (SD)	Data-sharing depth score, mean (SD)	Resource value score, mean (SD)	Resources present in, n	
Education/schools	22	3.09 (0.92)	2.82 (1.22)	3.05 (0.95)	45	
Public health (government)	16	2.94 (1.06)	2.62 (1.09)	2.88 (1.15)	30	
Academia/research	13	2.38 (1.04)	2.08 (1.04)	2.54 (1.05)	23	
Clinical health care	11	2.91 (0.94)	1.82 (0.87)	2.64 (0.92)	73	
Social and human services	11	3.27 (0.79)	2.64 (1.03)	3.09 (0.83)	27	
Multiple sectors	11	2.91 (0.94)	2.55 (1.04)	3.00 (1.00)	N/A^a^	
Organized government (tribal/local/state/federal) not included in others	10	2.90 (1.20)	2.70 (1.25)	3.00 (1.05)	33	
Mental/behavioral health care	10	3.00 (0.94)	2.50 (1.18)	2.70 (1.06)	33	
Information management infrastructure	9	2.78 (1.20)	3.11 (1.05)	2.78 (1.09)	22	
Public safety/law enforcement	7	3.43 (0.53)	1.71 (1.11)	2.43 (1.13)	22	
Housing and homelessness	6	2.33 (0.52)	1.83 (0.98)	2.50 (0.55)	11	
Business	5	3.20 (0.45)	2.20 (1.30)	3.40 (0.55)	17	
Health care payers	4	2.00 (0.82)	1.75 (0.96)	1.75 (0.96)	48	
Criminal justice/correctional facilities	4	3.00 (0.82)	2.75 (1.26)	3.00 (0.82)	10	
Justice system/courts	4	2.50 (0.58)	2.25 (0.50)	2.50 (0.58)	17	
Food and nutrition	3	3.33 (0.58)	2.67 (1.53)	3.00 (1.00)	5	
Banking/financial	3	2.00 (0.00)	2.33 (1.53)	2.00 (0.00)	7	
Legal/law firms	1	4 (N/A)	4 (N/A)	4 (N/A)	3	
Not expressly discussed in resource	1	2 (N/A)	4 (N/A)	3 (N/A)	1	
Other community-based, community action group	1	4 (N/A)	3 (N/A)	3 (N/A)	2	
Planning, economic, or community development	1	2 (N/A)	4 (N/A)	2 (N/A)	1	
Transportation/infrastructure	1	3 (N/A)	4 (N/A)	4 (N/A)	3	
Other	N/A	N/A	N/A	N/A	11	

^a^N/A: not applicable.

## Discussion

This is the first systematic study of publicly available resources on legal data-sharing issues. Publicly available resources are often the resources of first resort for practitioners, and the presence or absence of resources may factor in decisions to pursue a data-sharing project or engage with legal counsel. Consequently, it is important to understand what resources exist and what gaps are present. This paper helps map the existing landscape and can inform future work. For example, a number of quality resources exist for laws that govern health data, but fewer resources exist that discuss legal data-sharing issues pertaining to other social determinants of health, such as housing and homelessness.

It is possible that high numbers of resources addressing the same law might be an indicator of legal complexity or perceived legal barriers associated with that law. For example, HIPAA was one of the laws that was discussed in the most resources; however, HIPAA has generous exceptions that permit using data for public health and research purposes [6]. The fact that so many resources address HIPAA as a legal issue facing data sharing could be an indicator that the law is overly complex, misunderstood, or conservatively applied by organizations. Alternatively, the presence of a large number of publicly available resources addressing a law could indicate the law’s importance or significance to the activities of data custodians or simply a greater demand for knowledge and awareness of the law or legal issue.

Our findings suggest that good resources are difficult to find. Practitioners trying to find pertinent resources will have to sift through voluminous search results that are not useful to identify, understand, and address legal barriers to data sharing. Consequently, the difficulty of finding quality resources likely amplifies the perception of legal data-sharing barriers among practitioners.

To our surprise, the resources we identified cite nearly 100 different federal data-sharing laws or legal issues. The number of laws and legal issues was far higher than we expected. It suggests data-sharing challenges extend far beyond HIPAA, FERPA, and 42 CFR Part 2. Moreover, we also identified a large number of resources addressing multiple data protection laws or multiple sectors. These findings suggest that practitioners are working to address cross-sectoral legal data-sharing challenges. Given the patchwork legal data protection framework that exists in the United States, data-sharing projects designed to address the social determinants of health will likely cross multiple sectors and implicate the different data protection laws associated with those different sectors. The data silos—reinforced by these different data protection laws—have been cited as a barrier to the study of social determinants of health [2,4]. Our findings suggest that addressing these cross-sectoral challenges could be driving the development of publicly available legal resources. These challenges could be addressed with a comprehensive data-sharing framework [2,8]. For example, the European General Data Protection Regulation provides a straightforward legal analysis for cross-sectoral data sharing because it provides a common set of legal definitions, rules, and exceptions for all data controllers and custodians; in contrast, the United States has up to 6 different privacy laws that could apply to veterans’ health information [8,40]. Although privacy scholars have long cited the need for a comprehensive privacy law in the United States, Congress has struggled to appease the broad and diverse stakeholders for a national privacy law [8,41].

Our findings also suggest that many publicly available documents focus on legal agreements and consent documents that enable data sharing. Developing legal agreements from scratch can be incredibly expensive given the cost of legal services. Good template agreements can reduce costs tremendously and can be valuable starting points for legal counsel. Given these considerations, it is understandable that so many publicly available resources would address legal data-sharing agreements and consent documents. However, the template agreements we found varied in quality and utility. For example, some template agreements contained annotations that explained the purpose or function of specific terms and provisions [42,43], while some did not [44-46]. These annotations are useful to ensuring that agreements are well tailored to the needs of the data project. Without these annotations, there is a risk of contracting parties relying on a sample agreement that can inadvertently include counterproductive terms as boilerplate language.

We also note that we identified an interesting trend in publications over time. Our data show an increase in the number of publicly available resources relating to legal data-sharing issues from 2010 (ie, the earliest date within our scope) to 2018 but then a sudden decrease in 2019. It is possible that the increase in publications could be driven by the implementation of new data-sharing legislation and related regulations—like the federal Health Information Technology for Economic and Clinical Health Act (HITECH) Act (2009) and the 21st Century Cures Act (2016)—which created new data-sharing legal tools and opportunities. Some of the observed decrease after 2018 could be because our September 2019 search did not include October, November, and December 2019 publications; however, this likely does not explain the drop in the publication rate between 2018 and 2019 (from 2.75 publications per month to 1.67 publications per month). The decrease could be explained by an increase in interest in the newly implemented General Data Protection Regulation (GDPR) in the European Union, which affected many privacy policies of domestic entities and organizations due to its broad scope. Any resources that solely addressed GDPR legal issues would have been excluded from this research as an international law. If organizations that create publicly available resources on privacy shifted focus—and their finite resources—to the GDPR after its implementation, this could explain the sudden drop in publications that we observed.

Although publicly available resources can be very useful to practitioners in overcoming data-sharing barriers, there are several limitations associated with these resources. A Google search of “privacy” yields several trillion results, but only the top 400 or so are viewable under Google’s propriety platform. This is one example of the limitations inherent to systematically searching for publicly available resources using a propriety—and nontransparent—system. We took efforts to ensure that we sampled a broad range of this space, but these finding cannot be considered comprehensive. Moreover, publicly available resources are not necessarily permanent. Some highly rated resources identified in our search were later found to be unavailable in their original online locations [47], and quality resources, previously known to the authors, were not identified in this search [18]. A small number of our identified resources were updated prior to our completion of coding (ie, in 2020). Additionally, organizations may move or remove online resources, and we found this to be a common issue during our study. Thus, it can be difficult for practitioners to maintain an existing list of online resources. This highlights the need to develop a comprehensive and dynamic knowledge base that will compile and maintain publicly available data-sharing resources. For example, the resources identified in this study are now incorporated into the DASH Knowledge Base, an online, practitioner-focused database of data-sharing resources that includes tools to search for relevant and useful resources [24].

Additionally, our efforts to broadly sample this space (ie, through 75 separate searches) might have introduced some bias. For example, one of our search terms was “consent,” which might have inflated the number of template data-sharing forms that we found.

Finally, we note that this search does not include resources published in 2020, 2021 or 2022, so our results do not include the temporary emergency actions impelled by the COVID-19 response. Although this is a limitation, we note that the pace of federal legislation in data protection is glacial [8]. To our knowledge, no federal data protection act has been passed by Congress since the 21st Century Cures Act in 2016 (although the 2020 Coronavirus Aid, Relief, and Economic Security [CARES] Act included funding for loosely defined data modernization efforts).

This is the first systematic study of publicly available resources on legal data-sharing issues. Our findings describe the existing landscape of publicly available resources addressing legal data-sharing issues and can help identify future needs. We found existing gaps—like the Juvenile Justice and Delinquency Prevention Act which was discussed in only 1 resource or medical-legal partnerships, which was discussed in only 2 resources—where a lack of existing resources covering certain laws or applications allows existing data-sharing uncertainties to persist. We also found existing areas of saturation where certain laws and applications are covered extensively (eg, HIPAA and FERPA), such that new resource development might prove wasteful or perhaps even bury high quality resources deeper in search results. Moreover, many resources that we identified addressed multiple sectors or data protection laws, possibly indicating that cross-sectoral data sharing is a current priority in health informatics. Nevertheless, the volume of resources we found is an indicator that real and perceived legal issues are a substantial barrier to efforts to leverage data from different sectors to promote health. Although many resources exist to help practitioners navigate these legal issues, good resources may be hard to find.

## Appendix

Multimedia Appendix 1 Protocol.

Multimedia Appendix 2 Supplementary tables.

## Abbreviations

CARES: Coronavirus Aid, Relief, and Economic Security
CFR: Code of Federal Regulations
DASH: Data Across Sectors for Health
FERPA: Family Educational Rights and Privacy Act
HIPAA: Health Insurance Portability and Accountability Act
HITECH: Health Information Technology for Economic and Clinical Health Act
HUD: US Department of Housing and Urban Development
GDPR: General Data Protection Regulation
NCHS: National Center for Health Statistics
