# Supplementary material for: A Study of Publicly Available Resources Addressing Legal Data-Sharing Barriers: Systematic Assessment
Source: J Med Internet Res. 2022 Sep 6;24(9):e39333. doi: 10.2196/39333 (PMC9490527; doi:10.2196/39333)
Supplement: Multimedia Appendix 1 [file jmir_v24i9e39333_app1.docx]

#

# **PROTOCOL:**

## **Project Objective**

Identify, curate, and annotate resources to help professionals and other members of the public quickly identify relevant quality resources that discuss legal issues associated with data sharing and use.

## **Secondary Objectives**

1. Identify gaps in existing resources relating to data sharing and use
2. Identify legal issues that are highly saturated with available resources
3. Enhance understanding of the US data protection framework

## **Scoping Statement**

This collection is narrowly focused on those resources that address real and perceived legal barriers to data sharing with particularity. This includes resources discussing legal duties and exceptions as well as legal tools, agreements, and instruments that can facilitate data release and sharing in the US.

## **Scoping**

Inclusion/Exclusion Criteria

- Include:
  - Publicly available documents, toolkits, and resources
  - Federal laws or legal barriers **must be described**, not simply referenced.
  - Any resource with model legislation
    - <http://www.workforcedqc.org/sites/default/files/files/10%205%20NSCdatapolicytoolkit_webFINAL.pdf>
    - Or recommended legislation
  - Publicly available journal articles should be tagged for later evaluation
  - Sample Data Use agreements intended to inform external organizations
- Exclude:
  - Not publicly available Journal articles
  - Resources with a **specific** focus unrelated to health
    - EX: <https://nces.ed.gov/pubs2013/2013802.pdf>
    - Consider inclusion if related to social determinants of health.
      1. Many resources will not use the word “health” but may discuss issues that are highly related to health.
         1. EX: education, early childhood outcomes, transportation, homelessness, research
    - Mark as consult requested.
  - Non-governmental documents which are narrowly focused on obtaining secondary data **research** and do not specifically focus on obtaining secondary data from at least one non-academic sector. Documents that describe other forms of data sharing in addition to research should be included.
  - Sources that only compile (or link to) other resources.
  - Resources that only define legal tools or laws.
    - <https://www.aisp.upenn.edu/resources/legal-agreements-and-other-supporting-documents/>
  - Resources published prior to 2010
    - <http://www.aisp.upenn.edu/wp-content/uploads/2015/03/BeyondHIPAAPrivacyRule_EnhancingPrivacy_ImprovingHealthThroughResearch_2009.pdf>
  - Legislative updates
    - <https://drive.google.com/drive/folders/1-82nsjFxB7di3cp-ch4rYu35v2gH7oS2>
  - Blogs, editorials, news articles, and other commentaries on the state of the law or data sharing.
  - Privacy statements for commercial sites.
  - Organization-specific policies
  - Wikipedia sources
  - Publicly available statute or regulation text without accompanying analysis, commentary, or annotation.
  - Duplicate
  - Template data use agreements for internal organization use
  - Judicial Opinions
  - Applications for federal programs where sharing for evaluation is a requirement

## **Coding Criteria**

This project is designed to help the public identify appropriate resources. The coding scheme was developed in cooperation with DASH and heavily based on the DASH data framework. Many of the elements of this framework are not aligned perfectly with the patchwork privacy framework in the US.

## **Guiding Principle for Coding:**

Coders should apply codes that will help the public find resources that are relevant to them and avoid resources that are irrelevant.

- **Depth of legal issues discussion**
  - Note: this question **does not** consider the “quality” of the discussion, or whether the discussion appears consistent with the referenced statutes, regulations, or related judicial interpretations.
  - 4 - Resources describes law or legal issue in depth **and** provides an **extended** discussion of how law or legal issue is applied in specific use cases
    - EX: <https://drive.google.com/file/d/1YSmZ0RKl6fhWOuzTLynEgV3fRfSZIz0_/view?pli=1>
    - Template or example legal agreements with extended annotations or explanations
    - Caution: Powerpoints presentations likely lack sufficient context to qualify as an “extended discussion”
  - 3 - Resource:
    - Describes law or legal issue in **depth**, **or**
    - **Identifies** laws or legal issues related to specific use cases and provides **some** legal discussion, **or**
    - Provides overview of many different laws or legal issues without specific use cases
    - Template or example legal agreements with limited annotations or explanations
  - 2 - Resource:
    - Provides **limited** description or discussion of a law or legal issue, or
    - Identifies laws or legal issues related to specific use cases and provides only **basic** information about the law
    - Template or example legal agreements without annotation or explanation
  - 1 - Discussion of law or legal issue is superficial or non-existent (consider scoping)
    - Ex: <http://aaude.org/system/files/documents/public/reference/data-sharing-confidentiality-rules.pdf> - Note: this resource scored a “1” on data sharing discussion.
  - NOTE: Scoring does not reflect accuracy or quality of legal analysis
- **Depth of Data-Sharing discussion Rubric**
  - This question evaluates the extent the resource covers strategies to initiate or maintain at least one type of data sharing activity.
  - 4 - Resources:
    - Describes data-sharing issues and strategies in depth **and** provides an **extended** discussion of how data-sharing issues and strategies are applied in specific use cases, or
    - Provides **limited** description or discussion of data-sharing issues and strategies, **and** provides links to additional resources
  - 3 - Resource:
    - Provides **limited** description or discussion of data-sharing issues **and** strategies, or
    - Describes data-sharing issues in **depth**, **or**
    - Describes data-sharing strategies in **depth**,
  - 2 - Resource identifies data-sharing issues or strategies related to specific use cases
    - EX: <http://www.astho.org/uploadedFiles/Programs/Preparedness/Public_Health_Emergency_Law/Public_Health_and_Schools_Toolkit/04-PHS%20Comparing%20F%20and%20H%20FS%20Final%203-12.pdf>
  - 1 - Discussion of data-sharing issues is superficial or non-existent
  - NOTE: Scoring does not reflect accuracy or quality of data-sharing discussion
- **Value of this resource for addressing legal barriers (Scoping)**
  - This question is focused on the value to non-expert users.
  - Criteria that should be considered when evaluating this question:
    - Resources should receive **higher** scores if they have the following characteristics/elements:
      1. In-depth legal discussion (see question above)
      2. In-depth data-sharing discussion (see question above)
      3. User-friendly
      4. Checklists or step-by-step approaches
      5. Template forms or agreements
      6. Comprehensiveness
      7. Discussion which provides express strategies for dealing with legal barriers.
      8. From relevant regulatory agency
    - Resources should receive **lower** scores if they have the following characteristics/elements:
      1. Limited discussion of law or legal barriers
      2. Only tangential discussion of law or legal barriers
      3. Limited context or discussion (e.g., bullet point Powerpoint presentation)
    - EX: <http://rpp.wtgrantfoundation.org/developing-data-sharing-agreements> - Note this resource scored a 3 for data sharing, a 3 for legal discussion, but it received a 4 for value because it is user friendly and provides guidance that can be translated to actionable steps.
- **Domains: Sectors***
  - This item indicates the location of data affected by laws of interest.
  - NOTE: Categories may not be mutually exclusive.
  - A sector should be “marked” if the document describes a person or entity from the sector receiving the data, releasing the data, or using data (including storage and maintenance).
  - Mark all sectors that apply.
- **Domains: Data Types**
  - This item indicates the type of data used, or released.
  - NOTE: Categories may not be mutually exclusive.
  - Please consider the following:
    - Documents that discuss **HIPAA** likely relate to the following data types:
      1. Administrative
      2. Clinical Health
      3. Service
    - Documents that discuss **education records** relate to the following data types:
      1. Service
    - The Community-generated data type relates to a non-governmental person, organization, or entity. This data type should not be “marked” if other data types are more appropriate.
- **Domains: Affected Population**
  - This item relates to populations that are the specific focus of a discussion of legal issues and data sharing.
  - Categories should be “marked” if they are the specific focus of the document.
  - Indicate “Not expressly discussed in resource” if the resource:
    - Does not mention specific populations, or
    - Discusses all age groups
- **Affected Population: Populations at risk for health equity gaps**
  - Categories should be “marked” if they are the specific focus of the document.
- **Use Case***
  - This item relates to how the data use is described in the document.
  - Indicate “Not expressly discussed in resource” if the resource does not provide details on data use
  - NOTE: Laws rarely, if ever, are this specific when discussing data use or release. **A use case should be “marked” when a resource describes a use or activity that *could* encompass the given use case.**
  - Documents that discuss “evaluation” **may** relate to the following codes:
    - Statistical analysis to look for useful patterns and relationships in the data set
    - Calculating and reporting of metrics, indicators and dashboards enabling group comparison and tracking of progress over time
  - Documents that discuss “reporting” **may** relate to the following codes:
    - Reporting functions that allow users to specify and generate reports using items from a menu
    - Calculating and reporting of metrics, indicators and dashboards enabling group comparison and tracking of progress over time
  - Documents that discuss integrated data systems **likely** relate to the following codes:
    - Using identifying information to match records across systems to create a more encompassing view of a person or case
- **Laws Described**
  - This item relates to only those laws that are described with particularity.
  - Only “mark” laws that are discussed in the context of data use or sharing.
  - Laws that are mentioned or identified only **should not** be “marked.”
  - The Other category may be used if unsure whether the discussed law is included in the provided list.
  - Note: Laws were added to this original list as they were described in the resource.
- **Legal Resources and Content***
  - Case Studies Applying Law
    - Mark if:
      1. Resource includes a case study of data use or sharing with **at least a limited discussion** of a legal strategy being employed or a legal issue being addressed.
      2. Resource includes a vignette describing data use or data sharing without discussion of law as applied in that case **if and only if** the vignette appears within a general discussion of the law
      3. Note: “Case Studies” are different than the “Use Cases” described in the “Depth of legal issues discussion” and “Depth of Data-Sharing discussion” items.
         1. Case Studies refers to specific entities using or sharing data
         2. Use cases refers to data use or data sharing activities generally.
  - Legal Agreements for Data Sharing
    - Mark if Resource
      1. includes template agreements
      2. Provides guidance relating to legal agreements, including consent documentation
  - Inter-Agency Data Sharing
    - Mark if discusses sharing between government agencies
  - Health Authority Use
  - Health Information Exchange
    - Mark if resource discusses the organizations that facilitate the sharing of electronic health information between healthcare providers. Note: Resources may discuss activities of HIEs beyond provider-provider sharing.
  - Resource Links
    - Mark if resource includes links to other resources discussing law or legal issues.
    - NOTE: resource must be within the scope (i.e., resource must discuss law or legal issue with particularity)
  - Provider Sharing
    - Relates to Healthcare providers. May or may not include Health Information Exchanges (see above)
  - Statistical Methods for Protecting Privacy and Confidentiality
    - May include statistical de-identification, chaffing, differential privacy, etc.
  - General Legal Overview (i.e., no specific application indicated)
    - Mark if the resource describes the law in broad terms.
    - This code is appropriate for general legal overviews that occur within some resources describing specific applications if the overview is sufficiently separated from the discussion of the specific application
      1. EX: general legal overview is included as an appendix to the document.
  - Frequently Asked Questions About Law
  - Data Sharing for Program Evaluation
  - Medical-Legal Partnerships
    - Note this is a term of art. It refers to cooperation between a healthcare provider and a legal counsel to address the root cause of illness or injury of the patient. This typically **does not** refer to sharing between healthcare providers and law enforcement.
  - Working with Legal Counsel
    - Mark if resource provides guidance to help individuals work with their organization’s legal counsel.
  - Consent (obtaining consent, waiving requirements, models)
    - Mark if resource includes discussion of consent, including, obtaining consent, waiving consent requirements, different consent models, or provides template consent forms.
  - Model Legislation
