# Supplementary material for: A Study of Publicly Available Resources Addressing Legal Data-Sharing Barriers: Systematic Assessment
Source: J Med Internet Res. 2022 Sep 6;24(9):e39333. doi: 10.2196/39333 (PMC9490527; doi:10.2196/39333)
Supplement: Multimedia Appendix 2 [file jmir_v24i9e39333_app2.docx]

**Supplemental Figure and Tables**

**Supplemental Figure 1. Number of resources in terms of (A) number of laws discussed, (B) number of sectors discussed (n=154)**

|  |  |
| --- | --- |

**Supplemental Table 1. List of resources: citation, sectors, legal depth, data sharing depth and resource value (n=154)**

| **Resource** | **Cited resource** | **Main sector** | **All sectors** | **Legal depth** | **Data sharing depth** | **Resource value** | |
| --- | --- | --- | --- | --- | --- | --- | --- |
| 1 | Military Health System. Submit a data sharing application. https://health.mil/military-health-topics/privacy-and-civil-liberties/submit-a-data-sharing-application?type=policies. Accessed Sep 09, 2019 | Health Care Payers | Clinical Health Care, Health Care Payers | 3 | 3 | 3 | |
| 2 | Results for America. 2018 state standard of excellence - data policies / agreements. https://2018state.results4america.org/state-standard-of-excellence/data-policies--agreements.html. Accessed Jan 15, 2020 | Organized Government (Tribal/Local/State/Federal) not included in others | Education / Schools,  Social and Human Services,  Tribal / Local / State / Federal Government- Organized government (bureaucracy) not included in others | 1 | 1 | 2 | |
| 3 | Medicaid and CHIP Payment and Access Commission (MACPAC). 42 CFR part 2 substance use disorder confidentiality regulations – implications for care integration in Medicaid. https://www.macpac.gov/wp-content/uploads/2018/01/ 42-cfr-part-2-substance-use-disorder-treatment-and-integration.pdf. Accessed Nov 19, 2019 | Mental / Behavioral Health Care | Clinical Health Care,  Health Care Payers,  Information Management Infrastructure,  Mental / Behavioral Health Care | 2 | 1 | 1 | |
| 4 | ISAO Standards Organization. A framework for state-level information sharing and analysis organizations. https://www.isao.org/storage/2018/06/isao-600-1-a-framework-for-state-level-isaos.pdf. Accessed Mar 05, 2020 | Information Management Infrastructure | Information Management Infrastructure,  Public Safety / Law Enforcement,  Tribal / Local / State / Federal Government- Organized government (bureaucracy) not included in others | 1 | 4 | 1 | |
| 5 | National League of Cities, Stewards of Change. A guide to building integrated data systems compatible with federal privacy laws. http://www.nlc.org/sites/ default/files/2016-12/data%20sharing%20for%20better%20results.pdf. Accessed Jan 24, 2020 | Multiple sectors | Clinical Health Care,  Criminal Justice - Correctional facilities (jails/prisons),  Education / Schools,  Food and Nutrition,  Housing & Homelessness,  Justice System / Courts,  Mental / Behavioral Health Care,  Social and Human Services | 3 | 3 | 3 | |
| 6 | DASH. A legal approach to sharing health & education data. http://dashconnect.org/wp-content/uploads/2018/05/dash-bright-spot_chicago.pdf. Accessed Sep 25, 2019 | Education / Schools | Education / Schools,  Public Health (Government) | 3 | 4 | 4 | |
| 7 | Pacific Northwest National Laboratory. A nonproliferation third party for dual-use industries-legal issues for consideration. https://www.pnnl.gov/main/ publications/external/technical_reports/pnnl-21908.pdf. Accessed Sep 10, 2019 | Public Safety / Law Enforcement | Business,  Public Safety / Law Enforcement | 3 | 4 | 4 | |
| 8 | American Probation and Parole Association. A note on HIPAA and 42 CFR part 2. https://www.appa-net.org/eweb/docs/appa/pubs/dmjhis.pdf. Accessed Sep 25, 2019 | Criminal Justice / Correctional facilities | Clinical Health Care,  Criminal Justice - Correctional facilities (jails/prisons),  Health Care Payers,  Mental / Behavioral Health Care | 3 | 3 | 3 | |
| 9 | eGEMs. A solutions-based approach to building data-sharing partnerships. https://egems.academyhealth.org/articles/10.5334/egems.236/. Accessed Sep 09, 2019 | Academia / Research | Academia / Research | 1 | 4 | 4 | |
| 10 | EPA. Access to toxic substances control act confidential business information: a guide for access to TSCA CBI for state local and tribal governments. https://nepis.epa.gov/exe/zypurl.cgi?dockey=p100uwsj.txt. Accessed Jan 15, 2020 | Organized Government (Tribal/Local/State/Federal) not included in others | Business, Public Health (Government), Tribal / Local / State / Federal Government- Organized government (bureaucracy) not included in others | 4 | 3 | 4 | |
| 11 | UC Berkeley Center for Healthcare Organizational + Innovation Research (CHOIR). Accountable communities for health: data-sharing toolkit. http://cachi.org/uploads/resources/ach-data-sharing-toolkit-Dec-2016.pdf. Accessed Nov 20, 2019 | Planning, Economic, or Community Development - | Other Community-based - Community action group,  Planning / Economic or Community Development,  Tribal / Local / State / Federal Government- Organized government (bureaucracy) not included in others | 2 | 4 | 2 | |
| 12 | California Healthcare Foundation. Achieving the right balance: privacy and security policies to support electronic health information exchange. https://www.chcf.org/wp-content/uploads/2017/12/pdf-acheivingbalanceprivacysecurityhie.pdf. Accessed Nov 19, 2019 | Information Management Infrastructure | Clinical Health Care,  Health Care Payers,  Information Management Infrastructure | 1 | 3 | 2 | |
| 13 | MAPC (Metropolitan Area Planning Council). Addressing the opioid epidemic: information sharing toolkit. https://www.mapc.org/resource-library/addressing-the-opioid-epidemic-information-sharing-toolkit/. Accessed Sep 25, 2019 | Mental / Behavioral Health Care | Clinical Health Care,  Education / Schools,  Health Care Payers,  Mental / Behavioral Health Care | 4 | 1 | 2 |  |
| 14 | National Academy of Education. Advancing educational research and student privacy in the “big data” era. https://naeducation.org/wp-content/uploads/ 2017/05/ho-final.pdf. Accessed Jan 24, 2020 | Education / Schools | Academia / Research,  Business,  Education / Schools | 3 | 4 | 3 |  |
| 15 | Council of Large Public Housing Authorities (CLPHA). Aligning education and housing: data sharing agreement template. https://housingis.org/content/aligning-education-and-housing-data-sharing-agreement-template. Accessed Nov 18, 2019 | Housing & Homelessness | Education / Schools,  Housing & Homelessness | 3 | 1 | 3 |  |
| 16 | The Council of Large Public Housing Authorities. Aligning education and housing: data sharing agreement template for intermediary organizations. https://nche.ed.gov/wp-content/uploads/2018/11/clpha-data-template-io.pdf. Accessed Nov 18, 2019 | Housing & Homelessness | Education / Schools,  Housing & Homelessness | 3 | 1 | 3 |  |
| 17 | ASTHO. Authorities and limitations in sharing information between public health agencies and law enforcement. https://www.astho.org/programs/ preparedness/public-health-emergency-law/public-health-and-information-sharing-toolkit/authorities-and-limitations/. Accessed Dec 11, 2019 | Public Health (Government) | Public Health (Government),  Public Safety / Law Enforcement | 4 | 4 | 4 |  |
| 18 | Integrated Behavioral Health Partners. Behavioral health data sharing toolkit. http://www.ibhpartners.org/get-started/behavioral-health-data-sharing-toolkit/. Accessed Sep 06, 2019 | Mental / Behavioral Health Care | Clinical Health Care,  Mental / Behavioral Health Care,  other (cs): specific to California | 4 | 4 | 4 |  |
| 19 | Mass eHealth Institute. Behavioral health information sharing. https://mehi.masstech.org/support/learning-collaboratives/behavioral-health-information-sharing. Accessed Jan 08, 2020 | Mental / Behavioral Health Care | Clinical Health Care,  Health Care Payers,  Information Management Infrastructure,  Mental / Behavioral Health Care | 4 | 4 | 4 |  |
| 20 | Bureau of Justice Statistics, Office of Justice Programs, DOJ. Bureau of justice statistics data protection guidelines. https://www.bjs.gov/content/pub/pdf/ bjs_data_protection_guidelines.pdf. Accessed Sep 25, 2019 | Justice System / Courts | Academia / Research,  Criminal Justice - Correctional facilities (jails/prisons),  Justice System / Courts,  Public Safety / Law Enforcement,  Tribal / Local / State / Federal Government- Organized government (bureaucracy) not included in others | 3 | 2 | 3 |  |
| 21 | Multi-Regional Clinical Trials Center at Harvard University. Clinical trials data sharing. http://www.nationalacademies.org/hmd/~/media/files/activity%20files/ research/data-sharing/mtg2/46_mark%20barnes_ppt_4feb14.pdf. Accessed Sep 25, 2019 | Academia / Research | Academia / Research,  Clinical Health Care | 2 | 2 | 1 |  |
| 22 | Association of State and Territorial Health Officials. Comparison of FERPA and HIPAA privacy rule for accessing student health data. http://www.astho.org/ programs/preparedness/public-health-emergency-law/public-health-and-schools-toolkit/comparison-of-FERPA-and-HIPAA-privacy-rule/. Accessed Sep 25, 2019 | Education / Schools | Clinical Health Care,  Education / Schools,  Health Care Payers | 3 | 1 | 3 |  |
| 23 | University of Chicago Legal Forum. Confidentiality agreements in the administrative state. https://chicagounbound.uchicago.edu/cgi/ viewcontent.cgi?article=1611&context=uclf. Accessed Mar 03, 2020 | Business | Business,  Tribal / Local / State / Federal Government- Organized government (bureaucracy) not included in others | 3 | 1 | 3 |  |
| 24 | School of Government, UNC-Chapel Hill. Confidentiality and information sharing (chapter 14 of the manual titled, abuse, neglect, dependency, and termination of parental rights). https://www.sog.unc.edu/sites/www.sog.unc.edu/files/book_chapter/14%20confidentiality%20and%20info%20sharing.pdf. Accessed Apr 28, 2020 | Social and Human Services | Clinical Health Care,  Criminal Justice - Correctional facilities (jails/prisons),  Education / Schools,  Health Care Payers,  Justice System / Courts,  Mental / Behavioral Health Care,  Social and Human Services | 4 | 1 | 3 |  |
| 25 | Unemployment and training administration, DOL. Conformity requirements for state UC laws. https://oui.doleta.gov/unemploy/pdf/uilaws_confidentiality.pdf. Accessed Jan 29, 2020 | Social and Human Services | Social and Human Services,  Tribal / Local / State / Federal Government- Organized government (bureaucracy) not included in others | 3 | 1 | 2 |  |
| 26 | Office of Policy and Planning, Office of the National Coordinator for Health IT. Consumer consent options for electronic health information exchange: policy considerations and analysis. http://www.jhconnect.org/resource/consumer-consent-options-for-electronic-health-information-exchange-policy-considerations-and-analysis. Accessed Nov 19, 2019 | Information Management Infrastructure | Clinical Health Care,  Health Care Payers,  Information Management Infrastructure,  Mental / Behavioral Health Care | 4 | 3 | 4 |  |
| 27 | Alameda County Behavioral Health Care Services. Court dependents sharing confidential mental health information. http://www.acbhcs.org/providers/qi/docs/ training/2012/info_sharing_presentations.pdf. Accessed Sep 06, 2019 | Mental / Behavioral Health Care | Clinical Health Care,  Education / Schools,  Justice System / Courts,  Mental / Behavioral Health Care,  Social and Human Services | 3 | 2 | 3 |  |
| 28 | Congressional Research Service. Cross-border data sharing under the cloud act. https://fas.org/sgp/crs/misc/r45173.pdf. Accessed Apr 27, 2020 | Public Safety / Law Enforcement | Public Safety / Law Enforcement,  Tribal / Local / State / Federal Government- Organized government (bureaucracy) not included in others | 4 | 2 | 3 |  |
| 29 | Rochester Health Information Organization (RHIO). Cross-sector data exchange: data use and legal privacy. https://rhioportal.grrhio.org/pages/ getmediafile.aspx?fileid=3549559. Accessed Nov 19, 2019 | Multiple sectors | Banking/Financial, Clinical Health Care, Education / Schools, Health Care Payers, Housing & Homelessness, Information Management Infrastructure, Legal / Law Firms, Mental / Behavioral Health Care, Public Safety / Law Enforcement, Social and Human Services, Transportation / Infrastructure | 3 | 3 | 4 |  |
| 30 | NYC Administration of Children's Services. Crossover youth consent to share confidential information. https://www1.nyc.gov/assets/acs/pdf/cypm/ cypm_consent_form_final_12_31_14_english.pdf. Accessed Nov 14, 2019 | Criminal Justice / Correctional facilities | Criminal Justice - Correctional facilities (jails/prisons),  Education / Schools,  Mental / Behavioral Health Care,  Social and Human Services | 2 | 1 | 2 |  |
| 31 | Congressional Research Service. Cybersecurity and information sharing: legal challenges and solutions. https://fas.org/sgp/crs/intel/r43941.pdf. Accessed Dec 18, 2019 | Organized Government (Tribal/Local/State/Federal) not included in others | Business,  Public Safety / Law Enforcement,  Tribal / Local / State / Federal Government- Organized government (bureaucracy) not included in others | 4 | 4 | 4 |  |
| 32 | DHS; DOJ. Cybersecurity information sharing act – frequently asked questions. https://www.us-cert.gov/sites/default/files/ais_files/cisa_faqs.pdf. Accessed Dec 06, 2019 | Public Safety / Law Enforcement | Public Safety / Law Enforcement,  Tribal / Local / State / Federal Government- Organized government (bureaucracy) not included in others | 3 | 1 | 3 |  |
| 33 | US Department of Education. Data-sharing tool kit for communities: how to leverage community relationships while protecting student privacy. https://www2.ed.gov/programs/promiseneighborhoods/datasharingtool.pdf. Accessed Nov 20, 2019 | Education / Schools | Education / Schools | 4 | 4 | 4 |  |
| 34 | Office of Superintendent of Public Instructions, WA. Data & reporting: protecting student privacy. https://www.k12.wa.us/data-reporting/protecting-student-privacy. Accessed Dec 11, 2019 | Education / Schools | Academia / Research,  Education / Schools | 1 | 2 | 1 |  |
| 35 | Center for children and the law, American bar association. Data and information sharing (children). http://www.fostercareandeducation.org/areasoffocus/ datainformationsharing.aspx. Accessed Jan 24, 2020 | Social and Human Services | Education / Schools,  Social and Human Services | 4 | 4 | 4 |  |
| 36 | Strive Together. Data drives school-community collaboration. http://circ.communityreport.org/data-sharing-playbook/reports/73/data-drives-school-community-collaboration#page-1. Accessed Jan 08, 2020 | Education / Schools | Education / Schools | 2 | 3 | 2 |  |
| 37 | Dept of Education, Louisiana. Data governance and student privacy. https://www.louisianabelieves.com/resources/library/data-center/protecting-student-privacy. Accessed Jan 24, 2020 | Education / Schools | Education / Schools | 3 | 1 | 3 |  |
| 38 | Medicaid Innovation Accelerator Program. Data privacy, data use, and data use agreements. https://www.medicaid.gov/state-resource-center/innovation-accelerator-program/iap-downloads/program-areas/dua-factsheet.pdf. Accessed Jan 09, 2020 | Health Care Payers | Health Care Payers,  Public Health (Government),  Tribal / Local / State / Federal Government- Organized government (bureaucracy) not included in others | 1 | 1 | 1 |  |
| 39 | FEMA. Data sharing agreement content. https://www.FEMA.gov/media-library-data/1416582805604-99d579555db42353486e5665506780c8/data-sha.pdf. Accessed Jan 09, 2020 | Public Health (Government) | Public Health (Government),  Tribal / Local / State / Federal Government- Organized government (bureaucracy) not included in others | 4 | 2 | 4 |  |
| 40 | DATA ACROSS SECTORS FOR HEALTH (DASH). Data sharing and the law: deep dive on consent. http://dashconnect.org/wp-content/uploads/2018/11/data-sharing-and-the-law-deep-dive-on-consent.pdf. Accessed Sep 06, 2019 | Multiple sectors | Clinical Health Care,  Education / Schools,  Mental / Behavioral Health Care,  Public Health (Government) | 3 | 3 | 4 |  |
| 41 | Dept. of Administration, State of Minnesota. Data sharing between law enforcement and schools. https://mn.gov/admin/data-practices/data/types/ education/law-enforcement-schools/. Accessed Nov 19, 2019 | Multiple sectors | Education / Schools, Justice System / Courts, Public Safety / Law Enforcement | 3 | 2 | 3 |  |
| 42 | State Data Sharing Initiative. Legal guide to administrative data sharing for economic and workforce development. http://www.statedatasharing.org/data-sharing/. Accessed Apr 27, 2020 | Organized Government (Tribal/Local/State/Federal) not included in others | Tribal / Local / State / Federal Government- Organized government (bureaucracy) not included in others | 4 | 4 | 4 |  |
| 43 | Ohio Dept. of Education. Data sharing for program evaluation. https://education.ohio.gov/getattachment/topics/research-evaluation-and-advanced-analytics/sections/our-work-includes/data_sharing_guidance _for_districts.pdf.aspx?lang=en-us. Accessed Dec 11, 2019 | Education / Schools | Clinical Health Care,  Education / Schools | 4 | 4 | 4 |  |
| 44 | Coalition for Access and Opportunity. Data Sharing in Public Benefit Programs: An Action Agenda for Removing Barriers. https://www.singlestopusa.org/wp-content/uploads/2012/11/data_sharing_in_public_benefit_programs_11_2_12.pdf. Accessed Sep 01, 2020 | Social and Human Services | Clinical Health Care,  Health Care Payers,  Social and Human Services | 2 | 2 | 2 |  |
| 45 | NIH. Data sharing regulations/policy/guidance chart for NIH awards. https://grants.nih.gov/grants/policy/data_sharing/data_sharing_chart.doc. Accessed Apr 27, 2020 | Academia / Research | Academia / Research,  Clinical Health Care | 3 | 1 | 2 |  |
| 46 | HUD. Data sharing road map: improving student outcomes through partnerships between public housing agencies and school districts. https://www.hud.gov/ sites/documents/datasharingroadmap.pdf. Accessed Sep 25, 2019 | Education / Schools | Education / Schools,  Housing & Homelessness | 4 | 4 | 4 |  |
| 47 | Baylor college of medicine-Center for Medical Ethics and Health Policy. Data sharing, privacy, and academic research. https://sites.nationalacademies.org/cs/ groups/pgasite/documents/webpage/pga_169008.pdf. Accessed Sep 09, 2019 | Academia / Research | Academia / Research,  Clinical Health Care | 1 | 1 | 1 |  |
| 48 | Colorado Clinical and Translational Sciences Institute. Data sharing: creating agreements in support of community-academic partnerships. http://www.ucdenver.edu/research/cctsi/community-engagement/resources/ documents/datasharingcreatingagreements.pdf. Accessed Sep 03, 2019 | Other Community-based - Community action group | Academia / Research,  Other Community-based - Community action group | 4 | 3 | 3 |  |
| 49 | California Department of Education (“CDE”), the California Department of Social Services (“CDSS”), Bureau of Children’s Justice at the California Department of Justice (“DOJ”). Dear colleague letter: foster youth information-sharing. https://oag.ca.gov/sites/all/files/agweb/pdfs/bcj/fy-info.pdf. Accessed Nov 14, 2019 | Multiple sectors | Clinical Health Care,  Education / Schools,  Justice System / Courts,  Mental / Behavioral Health Care,  Social and Human Services | 4 | 1 | 3 |  |
| 50 | MDPH, Massachusetts Executive Office of Health and Human Services. Department of public health confidentiality procedures. https://www.mass.gov/files/documents/2016/07/rc/mdph-confidentiality-procedures.pdf. Accessed Mar 04, 2020 | Public Health (Government) | Clinical Health Care,  Health Care Payers,  Mental / Behavioral Health Care,  Public Health (Government) | 4 | 4 | 4 |  |
| 51 | Minnesota Department of Human Services. DHS data sharing framework for human services integration. https://www.dhs.state.mn.us/main/groups/ agencywide/documents/pub/dhs16_165323.pdf. Accessed Dec 19, 2019 | Social and Human Services | Clinical Health Care,  Health Care Payers,  Social and Human Services | 3 | 3 | 3 |  |
| 52 | Journal of Migration and Human Security. Enforcement, integration, and the future of immigration federalism (by Cristina Rodriguez, yale law school). https://journals.sagepub.com/doi/pdf/10.1177/233150241700500215. Accessed Apr 28, 2020 | Public Safety / Law Enforcement | Public Safety / Law Enforcement,  note: focused on immigration enforcement | 4 | 2 | 1 |  |
| 53 | National Association of Insurance Commissioners (NAIC). Ethical considerations in protecting confidential records and information sharing. https://naic.org/insurance_summit/documents/insurance_summit_2019_fr_14.pdf. Accessed Feb 06, 2020 | Health Care Payers | Banking/Financial,  Health Care Payers,  Tribal / Local / State / Federal Government- Organized government (bureaucracy) not included in others | 2 | 2 | 1 |  |
| 54 | DOJ. Exemption 7 of FIOA. https://www.justice.gov/oip/training/ exemption_7_july_2019/download. Accessed Jan 09, 2020 | Public Safety / Law Enforcement | Public Safety / Law Enforcement,  Tribal / Local / State / Federal Government- Organized government (bureaucracy) not included in others | 3 | 1 | 2 |  |
| 55 | North Carolina Health Information Exchange Authority. FAQ on NC health information exchange. https://hiea.nc.gov/frequently-asked-questions. Accessed Mar 03, 2020 | Information Management Infrastructure | Clinical Health Care,  Information Management Infrastructure | 3 | 4 | 2 |  |
| 56 | Federal Highway Administration. Federal highway administration information-sharing guidebook for transportation management centers, emergency operations centers, and fusion centers. https://ops.fhwa.dot.gov/publications/fhwahop09003/ tmc_eoc_guidebook.pdf. Accessed Apr 28, 2020 | Transportation / Infrastructure | Information Management Infrastructure,  Justice System / Courts,  Public Health (Government),  Public Safety / Law Enforcement,  Transportation / Infrastructure | 3 | 4 | 4 |  |
| 57 | CDC. Federal public health laws supporting data use and sharing. https://www.cdc.gov/phlp/docs/datasharing-laws.pdf. Accessed Sep 06, 2019 | Clinical Health Care | Clinical Health Care,  Public Health (Government) | 3 | 2 | 2 |  |
| 58 | National Association of Student Financial Aid Administrators (NASFAA). Financial aid data sharing. http://www.nasfaa.org/uploads/documents/ june_2019_data_sharing_white_paper.pdf. Accessed Sep 25, 2019 | Education / Schools | Banking/Financial, Education / Schools | 4 | 4 | 4 |  |
| 59 | Feeding America & Center for Health Law and Policy Innovation at Harvard Law School. Food banks as partners in health promotion: how HIPAA and concerns about protecting patient information affect your partnership. https://www.chlpi.org/wp-content/uploads/2013/12/food-banks-as-partners_HIPAA_Mar-2017.pdf. Accessed Nov 15, 2019 | Food and Nutrition | Clinical Health Care,  Food and Nutrition,  Health Care Payers | 4 | 4 | 4 |  |
| 60 | The Association of State and Territorial Health Officials (ASTHO). Foodborne disease outbreak response: assessing the legal and institutional framework for interagency information sharing. https://essentialelements.naccho.org/archives/8296. Accessed Nov 15, 2019 | Food and Nutrition | Food and Nutrition,  Public Health (Government) | 3 | 3 | 3 |  |
| 61 | FTC. Gramm-Leach-Bliley act. https://www.ftc.gov/tips-advice/business-center/privacy-and-security/gramm-leach-bliley-act. Accessed Sep 03, 2019 | Business | Banking/Financial | 3 | 1 | 3 |  |
| 62 | Health Resources and Services Administration, HHS. Guidance for donor and recipient information sharing. https://optn.transplant.hrsa.gov/resources/ guidance/guidance-for-donor-and-recipient-information-sharing/. Accessed Dec 18, 2019 | Clinical Health Care | Clinical Health Care | 3 | 3 | 3 |  |
| 63 | Financial Stability Board. Guidance on private sector information sharing. https://www.fsb.org/2017/11/guidance-on-private-sector-information-sharing/. Accessed Sep 10, 2019 | Banking/Financial | Banking/Financial,  Business,  Public Safety / Law Enforcement | 2 | 4 | 2 |  |
| 64 | NY Dept. of Health. Guidance to managed care organizations, health homes, care management agencies, and providers: sharing protected health information for outreach to support enrollment of individuals in health homes. https://www.health.ny.gov/health_care/medicaid/program/medicaid_health_homes/docs/guidance_to_mcos_hhs_cma_and_providers_re_info_sharing.pdf. Accessed Nov 20, 2019 | Clinical Health Care | Clinical Health Care,  Health Care Payers,  Mental / Behavioral Health Care,  Public Health (Government) | 4 | 3 | 4 |  |
| 65 | Office of Planning, Research and Evaluation, Administration for Children and Families, HHS. Guidelines for developing data sharing agreements to use state administrative data for early care and education research. https://www.acf.hhs.gov/sites/default/files/opre/guidelines_for_developing_data_sharing_agreements_508_7_16_18_508.pdf. Accessed Jan 15, 2020 | Social and Human Services | Education / Schools,  Social and Human Services | 2 | 2 | 2 |  |
| 66 | US district court - middle district North Carolina. Guidelines for the filing of confidential information in civil cases. https://www.ncmd.uscourts.gov/sites/ ncmd/files/guidelinesconfidential2014.pdf. Accessed Sep 10, 2019 | Justice System / Courts | Justice System / Courts | 2 | 2 | 2 |  |
| 67 | Privacy rights clearinghouse. Health information exchange and your privacy. https://privacyrights.org/consumer-guides/health-information-exchange-and-your-privacy-california-medical-privacy-series. Accessed Nov 19, 2019 | Information Management Infrastructure | Clinical Health Care,  Health Care Payers,  Information Management Infrastructure | 3 | 2 | 2 |  |
| 68 | CT legislative program review and investigations committee. Health information privacy in selected state programs. https://www.cga.ct.gov/pri/docs/2015/ health%20information%20privacy%20in%20selected%20state%20programs.pdf. Accessed Jan 09, 2020 | Public Health (Government) | Clinical Health Care,  Health Care Payers,  Public Health (Government) | 3 | 2 | 2 |  |
| 69 | HealthIT.gov by The Office of the National Coordinator for Health Information Technology (ONC). Health information privacy law and policy. https://www.healthit.gov/topic/health-information-privacy-law-and-policy. Accessed Sep 06, 2019 | Information Management Infrastructure | Clinical Health Care,  Health Care Payers,  Information Management Infrastructure | 2 | 1 | 3 |  |
| 70 | National Academies Press. Healthcare data as a public good: privacy and security (chapter 5 of the document "clinical data as the basic staple of health learning: creating and protecting a public good: workshop summary."). https://www.ncbi.nlm.nih.gov/books/nbk54293/. Accessed Apr 27, 2020 | Clinical Health Care | Academia / Research,  Clinical Health Care,  Health Care Payers | 1 | 3 | 1 |  |
| 71 | American Health Information Management Association (AHIMA). Hie management and operational considerations. https://library.ahima.org/doc?oid=105190#.xl7yyahkg-u. Accessed Mar 03, 2020 | Information Management Infrastructure | Clinical Health Care,  Health Care Payers,  Information Management Infrastructure,  Public Health (Government) | 4 | 4 | 4 |  |
| 72 | HHS. HIPAA for professionals - research. https://www.hhs.gov/HIPAA/for-professionals/special-topics/research/index.html. Accessed Mar 03, 2020 | Academia / Research | Academia / Research,  Clinical Health Care,  Health Care Payers | 3 | 1 | 3 |  |
| 73 | NASTAD. HIV data privacy and confidentiality: legal & ethical considerations for health department data sharing. https://www.nastad.org/sites/default/files/ uploads/2018/nastad-hiv-data-privacy-06062018.pdf. Accessed Feb 20, 2020 | Public Health (Government) | Clinical Health Care,  Health Care Payers,  Justice System / Courts,  Public Health (Government),  Public Safety / Law Enforcement | 3 | 3 | 3 |  |
| 74 | National Human Services Data Consortium. Housing and healthcare: partnerships for statewide data sharing. https://nhsdc.org/wp-content/uploads/2019/04/day2-session4-melodyb-mi-statewide-data-sharing.pdf. Accessed Nov 18, 2019 | Multiple sectors | Clinical Health Care,  Criminal Justice - Correctional facilities (jails/prisons),  Health Care Payers,  Housing & Homelessness,  Justice System / Courts,  Mental / Behavioral Health Care,  Public Health (Government),  Social and Human Services,  Tribal / Local / State / Federal Government- Organized government (bureaucracy) not included in others | 1 | 2 | 1 |  |
| 75 | HelathIT.gov by the Office of the National Coordinator for Health Information Technology (ONC). How HIPAA supports data sharing. https://www.healthit.gov/topic/interoperability/how-HIPAA-supports-data-sharing. Accessed Sep 06, 2019 | Clinical Health Care | Clinical Health Care,  Health Care Payers,  Public Health (Government) | 4 | 2 | 4 |  |
| 76 | NA. Human subjects research. https://www.genome.gov/about-genomics/policy-issues/human-subjects-research-in-genomics. Accessed Jan 08, 2020 | Academia / Research | Academia / Research,  Clinical Health Care | 2 | 3 | 3 |  |
| 77 | Minnesota Department of Health. Immunization data sharing, HIPAA, and MIIC. https://www.health.state.mn.us/people/immunize/miic/privacy/ HIPAA.html. Accessed Sep 06, 2019 | Public Health (Government) | Clinical Health Care,  Health Care Payers,  Information Management Infrastructure,  Public Health (Government) | 4 | 1 | 4 |  |
| 78 | Department of Health and Mental Hygiene, Maryland. Improving the exchange and coordination of care for Medicaid eligible individuals accessing specialty behavioral health services. https://mmcp.health.maryland.gov/documents/jcrs/ datasharingjcrfinal11-15.pdf. Accessed Jan 08, 2020 | Mental / Behavioral Health Care | Clinical Health Care,  Mental / Behavioral Health Care | 3 | 3 | 3 |  |
| 79 | Sharon Public Health Dept, MA. Influenza vaccine consent and screening form for children. https://www.sharon.k12.ma.us/cms/lib/ma02202341/centricity/ domain/59/combined%20flu%202018.pdf. Accessed Jan 09, 2020 | Public Health (Government) | Education / Schools,  Public Health (Government) | 1 | 1 | 2 |  |
| 80 | IJIS Institute, The Urban Institute. Information sharing between criminal justice and healthcare communities to enhance health and public safety. https://www.quantumunitsed.com/get-material.php?id=504. Accessed Mar 02, 2020 | Criminal Justice / Correctional facilities | Clinical Health Care,  Criminal Justice - Correctional facilities (jails/prisons),  Mental / Behavioral Health Care | 3 | 3 | 3 |  |
| 81 | National Center for MEDICAL-LEGAL PARTNERSHIP. Information sharing in medical-legal partnerships: foundational concepts and resources. https://medical-legalpartnership.org/wp-content/uploads/2017/07/information-sharing-in-mlps.pdf. Accessed Nov 14, 2019 | Legal / Law Firms | Clinical Health Care,  Legal / Law Firms | 4 | 4 | 4 |  |
| 82 | Education Development Center. Information sharing: case examples. http://informationsharing.promoteprevent.org/. Accessed Nov 14, 2019 | Education / Schools | Clinical Health Care,  Education / Schools,  Justice System / Courts,  Mental / Behavioral Health Care,  Social and Human Services,  Tribal / Local / State / Federal Government- Organized government (bureaucracy) not included in others | 4 | 4 | 4 |  |
| 83 | The Office for Victims of Crime Training and Technical Assistance Center, DOJ. Information sharing>professional and legal responsibilities of law enforcement and prosecutors. https://www.ovcttac.gov/taskforceguide/eguide/3-operating-a-task-force/32-information-sharing/professional-and-legal-responsibilities/. Accessed Mar 02, 2020 | Justice System / Courts | Clinical Health Care,  Justice System / Courts,  Legal / Law Firms,  Mental / Behavioral Health Care,  Social and Human Services,  Tribal / Local / State / Federal Government- Organized government (bureaucracy) not included in others | 3 | 3 | 3 |  |
| 84 | Council of State Governments Justice Center. Information sharing in criminal justice–mental health collaborations. https://www.bja.gov/publications/ csg_cjmh_info_sharing.pdf. Accessed Sep 25, 2019 | Criminal Justice / Correctional facilities | Clinical Health Care,  Criminal Justice - Correctional facilities (jails/prisons),  Justice System / Courts,  Mental / Behavioral Health Care,  Public Safety / Law Enforcement | 4 | 4 | 4 |  |
| 85 | CMS. Instructions for completing the data use agreement (DUA) form cms-r-0235 and the DUA. https://www.cms.gov/medicare/cms-forms/cms-forms/downloads/cms-r-0235.pdf. Accessed Jan 10, 2020 | Health Care Payers | Health Care Payers | 2 | 1 | 2 |  |
| 86 | Washington State Department of Commerce. Interagency data sharing agreement Washington state HMIS. http://www.commerce.wa.gov/wp-content/uploads/ 2016/07/hau-hmis-ids-agreenment-2-24-2016.pdf. Accessed Nov 18, 2019 | Housing & Homelessness | Housing & Homelessness,  Social and Human Services | 2 | 1 | 2 |  |
| 87 | DOL & ED. Joint guidance on data matching to facilitate WIOA performance reporting and evaluation. https://www2.ed.gov/policy/gen/guid/fpco/pdf/final-FERPA-tegl-report.pdf. Accessed Mar 03, 2020 | Education / Schools | Business,  Education / Schools | 4 | 2 | 3 |  |
| 88 | Connecticut DPH. Lead safe housing rule amendment supplemental materials. https://portal.ct.gov/dph/environmental-health/lead-poisoning-prevention-and-control/-/media/departments-and-agencies/dph/dph/environmental_health/lead/ circular_letters/2018/201808-dphs-response-to-the-lshr-amendment.pdf?la=en. Accessed Nov 18, 2019 | Housing & Homelessness | Housing & Homelessness,  Public Health (Government) | 2 | 2 | 2 |  |
| 89 | Milibank Quarterly. Legal barriers to the growth of health information exchange—boulders or pebbles? https://www.ncbi.nlm.nih.gov/pmc/articles/ pmc5835678/. Accessed Nov 21, 2019 | Clinical Health Care | Clinical Health Care,  Health Care Payers,  Information Management Infrastructure,  Mental / Behavioral Health Care | 4 | 1 | 2 |  |
| 90 | State Data Sharing Initiative. Legal guide to administrative data sharing for economic and workforce development. http://statedatasharing.org/data-sharing/2018-03_-_sds_legal_guide_to_administrative_data_sharing_for_ economic_and_workforce_development.pdf. Accessed Apr 27, 2020 | Business | Business,  Tribal / Local / State / Federal Government- Organized government (bureaucracy) not included in others | 4 | 4 | 4 |  |
| 91 | Council of State and Territorial Epidemiologists (CSTE). Legal issues concerning identifiable health data sharing between state/local public health authorities and tribal epidemiology centers in selected U.S. jurisdictions. http://www.cste2.org/webpdfs/legalissuesconcerningidentifiablehealthdatasharingbetweenstatelocalpublichealthauthoritiesandtribalepidemiologycentersinselectedusjurisdictionsfinal.pdf. Accessed Feb 20, 2020 | Public Health (Government) | Academia / Research,  Clinical Health Care,  Health Care Payers,  Public Health (Government),  Tribal / Local / State / Federal Government- Organized government (bureaucracy) not included in others | 4 | 4 | 4 |  |
| 92 | Actionable Intelligence for Social Policy. Legal issues for ids use: finding a way forward. https://www.aisp.upenn.edu/wp-content/uploads/2016/07/legal-issues.pdf. Accessed Sep 25, 2019 | Multiple sectors | Clinical Health Care,  Education / Schools,  Health Care Payers,  Housing & Homelessness,  Justice System / Courts,  Mental / Behavioral Health Care,  Public Safety / Law Enforcement,  Social and Human Services,  Tribal / Local / State / Federal Government- Organized government (bureaucracy) not included in others | 4 | 4 | 4 |  |
| 93 | Partnership for Public Health Law. Legal issues related to sharing of clinical health data with public health agencies. http://www.astho.org/public-policy/public-health-law/legal-issues-related-to-sharing-clinical-health-data-with-public-health-agencies/. Accessed Sep 06, 2019 | Public Health (Government) | Clinical Health Care,  Education / Schools,  Public Safety / Law Enforcement | 4 | 4 | 4 |  |
| 94 | California Integrated Data Exchange. Legal landscape for health information exchange. https://emsa.ca.gov/wp-content/uploads/sites/71/2017/07/legal-landscape-for-health-information-exchange.pdf. Accessed Nov 19, 2019 | Clinical Health Care | Clinical Health Care,  Health Care Payers,  Information Management Infrastructure,  Mental / Behavioral Health Care | 2 | 2 | 2 |  |
| 95 | ILLINOIS DEPARTMENT OF HUMAN SERVICES. Lifeline eligibility verification system - Illinois, DSA. https://www.dhs.state.il.us/onenetlibrary/ 27897/documents/initiatives/lifeline/idhs-levs-dsa-template-6-8-2018.pdf. Accessed Jan 10, 2020 | Multiple sectors | Business,  Clinical Health Care,  Food and Nutrition,  Health Care Payers,  Information Management Infrastructure,  Social and Human Services | 2 | 1 | 2 |  |
| 96 | Dept. of Labor and Industry, PA. Local workforce delivery system – memorandum of understanding. https://www.dli.pa.gov/businesses/workforce-development/documents/mou%20guidance.pdf. Accessed Jan 25, 2020 | Business | Business | 3 | 2 | 3 |  |
| 97 | Department of Education, LA. Louisiana’s data governance & student privacy guidebook. https://www.louisianabelieves.com/docs/default-source/data-management/student-privacy-planning-guide-(web).pdf?sfvrsn=19848c1f_16. Accessed Mar 04, 2020 | Education / Schools | Education / Schools | 3 | 3 | 3 |  |
| 98 | Healthdoers.org (site); HealthInfoNet & DASH (document). Maine’s homeless health information planning collaborative: recommendations for statewide HMIS & hie data integration. https://allin.healthdoers.org/wp-content/uploads/bp-attachments/8157/r-1.-maine-hie-hmis-collaborative-recommendations-2018.pdf. Accessed Nov 19, 2019 | Housing & Homelessness | Housing & Homelessness,  Information Management Infrastructure | 2 | 3 | 3 |  |
| 99 | Social Interest Solutions. Maximizing linkages: a policymaker’s guide to data sharing. https://www.socialinterest.org/wp-content/uploads/2019/04/ sis_maximizinglinkages_041919.pdf. Accessed Dec 11, 2019 | Social and Human Services | Health Care Payers,  Social and Human Services | 3 | 3 | 3 |  |
| 100 | SANS Institute. Medical data sharing: establishing trust in health information exchange. https://www.sans.org/reading-room/whitepapers/legal/medical-data-sharing-establishing-trust-health-information-exchange-37657. Accessed Sep 06, 2019 | Information Management Infrastructure | Clinical Health Care,  Information Management Infrastructure | 3 | 3 | 3 |  |
| 101 | Office of AIDS, California Dept. of Public Health. Methods and timeline for reporting HIV data: overview of legal requirements for providers, labs, and local health officers. http://paetc.org/wp-content/uploads/2017/12/data-sharing-logistical-requirements-11-20-17.pdf. Accessed Dec 12, 2019 | Public Health (Government) | Clinical Health Care,  Public Health (Government) | 3 | 2 | 3 |  |
| 102 | Common Sense Privacy Program. Navigate the privacy evaluation questions. https://privacy.commonsense.org/resource/full-evaluation-questions. Accessed Sep 25, 2019 | Business | Business,  Education / Schools | 3 | 3 | 4 |  |
| 103 | NCI. NIH genomic data sharing policy & grantee requirements. https://cancercontrol.cancer.gov/new_grantees/presentations/charlisse-caga-anan-data-sharing.pdf. Accessed Jan 09, 2020 | Academia / Research | Academia / Research | 1 | 2 | 1 |  |
| 104 | UNC Chapel Hill School of Government. North Carolina juvenile justice-behavioral health information sharing guide. https://www.sog.unc.edu/sites/ www.sog.unc.edu/files/information%20sharing%20guide%20final%20pdf%20to%20authors%202015-06-25.pdf. Accessed August 24, 2020 | Multiple sectors | Education / Schools,  Justice System / Courts,  Mental / Behavioral Health Care | 4 | 4 | 4 |  |
| 105 | NAIC Own Risk and Solvency Assessment. ORSA information sharing best practices. https://www.naic.org/documents/ cmte_e_orsai_sg_related_orsa_info_share_bp.pdf. Accessed Dec 18, 2019 | Banking/Financial | Banking/Financial,  Business | 2 | 1 | 2 |  |
| 106 | California Healthcare Foundation. Overcoming data-sharing challenges in the opioid epidemic: integrating substance use disorder treatment in primary care. https://www.chcf.org/wp-content/uploads/2018/07/ overcomingdatasharingchallengesopioid.pdf. Accessed Nov 15, 2019 | Mental / Behavioral Health Care | Clinical Health Care,  Health Care Payers,  Information Management Infrastructure,  Mental / Behavioral Health Care | 4 | 4 | 4 |  |
| 107 | FDA. Overview of commissioning and information sharing agreements with state and local government officials - FDA information paper. https://www.fda.gov/media/86166/download. Accessed Jan 25, 2020 | Food and Nutrition | Business, Food and Nutrition, Public Health (Government) | 3 | 1 | 2 |  |
| 108 | DOJ. Overview of the privacy act of 1974. https://www.justice.gov/opcl/ conditions-disclosure-third-parties. Accessed Mar 04, 2020 | Organized Government (Tribal/Local/State/Federal) not included in others | Public Safety / Law Enforcement,  Tribal / Local / State / Federal Government- Organized government (bureaucracy) not included in others | 4 | 1 | 3 |  |
| 109 | Stratis Health. Participation data sharing agreements. https://www.stratishealth.org/documents/healthit/homehealth/3.select/3-participation-data-sharing-agreements.doc. Accessed Nov 19, 2019 | Multiple sectors | Clinical Health Care,  Health Care Payers,  Information Management Infrastructure | 2 | 2 | 2 |  |
| 110 | Stratis Health and Minnesota Department of Health. Participation, data sharing, data use, business associate agreements for hie and EHR. https://www.stratishealth.org/documents/healthit/behavioralhealth/3.select/3-participation-data-sharing-data-use-business-associate-agreements-for-hie-and-ehr.docx. Accessed Apr 27, 2020 | Clinical Health Care | Clinical Health Care,  Health Care Payers,  Information Management Infrastructure | 2 | 1 | 3 |  |
| 111 | The Office of the National Coordinator for Health Information Technology (ONC). Patient consent for electronic health information exchange and interoperability. https://www.healthit.gov/topic/interoperability/patient-consent-electronic-health-information-exchange-and-interoperability. Accessed Sep 06, 2019 | Clinical Health Care | Clinical Health Care,  Health Care Payers | 3 | 1 | 2 |  |
| 112 | ASTHO. Pharmacy legal toolkit. https://astho.org/infectious-disease/pharmacy-legal-toolkit/. Accessed Dec 12, 2019 | Public Health (Government) | Clinical Health Care,  Public Health (Government) | 1 | 2 | 1 |  |
| 113 | California Cancer Registry. Policies and procedures for access to and disclosure of confidential data from the California cancer registry. https://www.ccrcal.org/ download/82/site-pdf-links/7430/ccrpoliciesprocedures_v05-2_3-19-2.pdf. Accessed Jan 09, 2020 | Public Health (Government) | Academia / Research,  Public Health (Government) | 2 | 2 | 2 |  |
| 114 | Mississippi state department of health. Principles and protocols for the release of health care data. https://msdh.ms.gov/msdhsite/_static/resources/3970.pdf. Accessed Feb 06, 2020 | Public Health (Government) | Academia / Research,  Clinical Health Care,  Public Health (Government) | 2 | 3 | 2 |  |
| 115 | DHS and DOJ. Privacy and civil liberties final guidelines: cybersecurity information sharing act of 2015. https://www.us-cert.gov/sites/default/files/ ais_files/privacy_and_civil_liberties_guidelines.pdf. Accessed Feb 06, 2020 | Public Safety / Law Enforcement | Public Safety / Law Enforcement,  Tribal / Local / State / Federal Government- Organized government (bureaucracy) not included in others | 3 | 1 | 3 |  |
| 116 | US Department of Education. Privacy and data sharing. https://studentprivacy.ed.gov/privacy-and-data-sharing. Accessed Sep 03, 2019 | Education / Schools | Academia / Research,  Education / Schools | 4 | 3 | 4 |  |
| 117 | STANFORD TECHNOLOGY LAW REVIEW. Privacy and synthetic datasets. https://www-cdn.law.stanford.edu/wp-content/uploads/2019/01/ bellovin_20190129-1.pdf. Accessed Apr 28, 2020 | Not expressly discussed in resource | Not expressly discussed in resource | 2 | 4 | 3 |  |
| 118 | FTC consumer information. Privacy choices for your personal financial information. https://www.consumer.ftc.gov/articles/0222-privacy-choices-your-personal-financial-information. Accessed Feb 20, 2020 | Banking/Financial | Banking/Financial,  Business | 2 | 2 | 2 |  |
| 119 | DHS. Privacy impact assessment for the law enforcement information sharing service (leis service). https://www.dhs.gov/sites/default/files/publications/ privacy-pia-ice-leiss-july2019_0.pdf. Accessed Mar 04, 2020 | Public Safety / Law Enforcement | Public Safety / Law Enforcement | 4 | 1 | 1 |  |
| 120 | California Dept. of Education. Privacy of student records collected and maintained by the California department of education. https://www.cde.ca.gov/ds/ed/dataprivacy.asp. Accessed Nov 15, 2019 | Education / Schools | Education / Schools | 2 | 1 | 2 |  |
| 121 | National immigration law center. Privacy protections in selected federal benefits programs. https://www.nilc.org/issues/economic-support/privacy-protections-in-selected-federal-benefits-programs/. Accessed Nov 15, 2019 | Social and Human Services | Social and Human Services | 3 | 3 | 3 |  |
| 122 | National Indian Child Welfare Association. Privacy, HIPAA, and information sharing fact sheet. https://www.nicwa.org/wp-content/uploads/2016/11/ 2014_privacyHIPAAandinfosharing_factsheet.pdf. Accessed Dec 18, 2019 | Multiple sectors | Health Care Payers,  Social and Human Services,  Tribal / Local / State / Federal Government- Organized government (bureaucracy) not included in others | 3 | 3 | 3 |  |
| 123 | Sunlight Foundation. Protecting data, protecting residents. https://sunlightfoundation.com/wp-content/uploads/2017/02/protecting-data-protecting-residents-whitepaper.pdf. Accessed Jan 25, 2020 | Organized Government (Tribal/Local/State/Federal) not included in others | Public Safety / Law Enforcement,  Tribal / Local / State / Federal Government- Organized government (bureaucracy) not included in others | 1 | 3 | 1 |  |
| 124 | Dept of Education. Protecting student privacy - data sharing. https://studentprivacy.ed.gov/content/data-sharing. Accessed Jan 24, 2020 | Education / Schools | Education / Schools | 4 | 4 | 4 |  |
| 125 | Association of state and territorial health officials (ASTHO). Public health collection, use, sharing, and protection of information. http://www.astho.org/programs/preparedness/public-health-emergency-law/public-health-and-information-sharing-toolkit/collection-use-sharing-and-protection-issue-brief/. Accessed Sep 06, 2019 | Public Health (Government) | Business,  Clinical Health Care,  Education / Schools,  Public Health (Government),  Public Safety / Law Enforcement,  Transportation / Infrastructure | 3 | 4 | 4 |  |
| 126 | The Network for Public Health Law. Public health information and data sharing: the legal framework. https://www.networkforphl.org/_asset/x59wj3/ webinar_public-health-information-and-data-sharing_8-18-16.pdf. Accessed Sep 11, 2019 | Public Health (Government) | Clinical Health Care,  Education / Schools,  Health Care Payers,  Mental / Behavioral Health Care,  Public Health (Government),  Tribal / Local / State / Federal Government- Organized government (bureaucracy) not included in others | 3 | 2 | 2 |  |
| 127 | Inter-university Consortium for Political and Social Research. Recommended informed consent language for data sharing. https://www.icpsr.umich.edu/icpsrweb/content/datamanagement/confidentiality/conf-language.html. Accessed Mar 04, 2020 | Academia / Research | Academia / Research | 4 | 3 | 4 |  |
| 128 | Data Quality Campaign. Roadmap for foster care and k–12 data linkages. http://2pido73em67o3eytaq1cp8au.wpengine.netdna-cdn.com/wp-content/ uploads/2017/02/dqc-foster-care-roadmap-02282017.pdf. Accessed Sep 25, 2019 | Education / Schools | Education / Schools,  Justice System / Courts,  Tribal / Local / State / Federal Government- Organized government (bureaucracy) not included in others | 2 | 4 | 2 |  |
| 129 | nationalservice.gov. Sample data sharing agreement. https://www.nationalservice.gov/sites/default/files/documents/appendix%20a.16%20-%20sample%20data%20sharing%20agreement.pdf. Accessed Jan 08, 2020 | Education / Schools | Education / Schools,  Social and Human Services | 2 | 1 | 2 |  |
| 130 | University of Kansas. Sample data use agreement, KU office of research. https://research.ku.edu/sites/rgs.drupal.ku.edu/files/docs/hscl_data_use_agree_07_13.doc. Accessed Jan 10, 2020 | Academia / Research | Academia / Research,  Clinical Health Care,  Health Care Payers | 2 | 1 | 3 |  |
| 131 | The Office of Community Oriented Policing Services, DOJ. School resource officers navigating information sharing. https://cops.usdoj.gov/html/dispatch/06-2013/sros_and_information_sharing.asp. Accessed Sep 25, 2019 | Education / Schools | Criminal Justice - Correctional facilities (jails/prisons),  Education / Schools | 4 | 3 | 4 |  |
| 132 | The National Academies Press. Sharing clinical research data: workshop summary (2013): chapter 3. https://www.nap.edu/read/18267/chapter/4. Accessed Sep 03, 2019 | Academia / Research | Academia / Research,  Clinical Health Care | 3 | 3 | 3 |  |
| 133 | DataONE. Sharing data: legal and policy considerations. https://www.dataone.org/best-practices/sharing-data-legal-and-policy-considerations. Accessed Dec 11, 2019 | Academia / Research | Academia / Research | 2 | 2 | 3 |  |
| 134 | SAMHSA. Sharing information across physical and behavioral health: debunking myths, developing strategies. https://www.integration.samhsa.gov/ integrated-care-models/sharing_information_across_physical_and_behaviroal_ health_webinar_slides.pdf. Accessed Nov 15, 2019 | Mental / Behavioral Health Care | Clinical Health Care,  Health Care Payers,  Information Management Infrastructure,  Mental / Behavioral Health Care | 2 | 2 | 2 |  |
| 135 | PLOS Biology. Sharing research data and intellectual property law: a primer. https://journals.plos.org/plosbiology/article?id=10.1371/journal.pbio.1002235. Accessed Sep 09, 2019 | Academia / Research | Academia / Research | 4 | 3 | 3 |  |
| 136 | Sunlight foundation. Sharing sensitive data within government. https://sunlightfoundation.com/2015/02/11/sharing-sensitive-data-within-government/. Accessed Sep 09, 2019 | Organized Government (Tribal/Local/State/Federal) not included in others | Academia / Research,  Tribal / Local / State / Federal Government- Organized government (bureaucracy) not included in others | 2 | 3 | 2 |  |
| 137 | The Network for Public Health Law. Snapshot: CIPSEA. https://www.networkforphl.org/_asset/rpm6et/snapshot-cipsea.pdf. Accessed Sep 06, 2019 | Organized Government (Tribal/Local/State/Federal) not included in others | Public Health (Government),  Tribal / Local / State / Federal Government- Organized government (bureaucracy) not included in others | 3 | 3 | 3 |  |
| 138 | State Data Sharing Initiative. State data sharing initiative tools: confidentiality laws & regulations database. http://www.statedatasharing.org/. Accessed Sep 25, 2019 | Organized Government (Tribal/Local/State/Federal) not included in others | Business,  Social and Human Services,  Tribal / Local / State / Federal Government- Organized government (bureaucracy) not included in others | 3 | 4 | 4 |  |
| 139 | FERPA SHERPA. State student privacy laws. https://FERPAsherpa.org/state-laws/. Accessed Sep 25, 2019 | Education / Schools | Education / Schools | 3 | 1 | 2 |  |
| 140 | Strive Together. Student data privacy: best practices. https://www.strivetogether.org/wp-content/uploads/2017/04/student-data-privacy-best-practices.pdf. Accessed Nov 14, 2019 | Education / Schools | Education / Schools | 3 | 3 | 3 |  |
| 141 | Medicaid and CHIP Payment and Access Commission. Substance use disorder confidentiality regulations and care integration in Medicaid. https://www.macpac.gov/wp-content/uploads/2018/03/substance-use-disorder-confidentiality-regulations-and-care-integration-in-medicaid-draft-chapter-and-recommendations.pdf. Accessed Sep 09, 2019 | Mental / Behavioral Health Care | Clinical Health Care,  Health Care Payers,  Mental / Behavioral Health Care | 2 | 2 | 2 |  |
| 142 | Journal of Law and Biosciences. Substitute consent to data sharing: a way forward for international dementia research? https://www.ncbi.nlm.nih.gov/ pmc/articles/pmc5570693/. Accessed Apr 27, 2020 | Academia / Research | Academia / Research,  Clinical Health Care | 3 | 1 | 2 |  |
| 143 | Office of Civil Rights, HHS. Summary of the HIPAA privacy rule. https://www.hhs.gov/HIPAA/for-professionals/privacy/laws-regulations/index.html. Accessed Mar 03, 2020 | Clinical Health Care | Clinical Health Care,  Health Care Payers | 3 | 1 | 3 |  |
| 144 | United States Government Accountability Office. Sustained and coordinated efforts could facilitate data sharing while protecting privacy. https://www.gao.gov/assets/660/652058.pdf. Accessed Dec 16, 2019 | Social and Human Services | Clinical Health Care,  Education / Schools,  Health Care Payers,  Mental / Behavioral Health Care,  Social and Human Services,  Tribal / Local / State / Federal Government- Organized government (bureaucracy) not included in others | 4 | 4 | 4 |  |
| 145 | Prepared by National eHealth Collaborative for SAMHSA - HRSA. The current state of sharing behavioral health information in health information exchanges. https://www.integration.samhsa.gov/operations-administration/ hie_paper_final.pdf. Accessed Nov 19, 2019 | Information Management Infrastructure | Clinical Health Care,  Health Care Payers,  Information Management Infrastructure,  Mental / Behavioral Health Care | 4 | 4 | 4 |  |
| 146 | Privacy Rights Clearinghouse. The HIPAA privacy rule: how may covered entities use and disclose health information. https://privacyrights.org/consumer-guides/HIPAA-privacy-rule-how-may-covered-entities-use-and-disclose-health-information. Accessed Nov 18, 2019 | Clinical Health Care | Clinical Health Care,  Health Care Payers | 3 | 1 | 3 |  |
| 147 | Urban Libraries Council. The leaders library card challenge success strategy. https://www.urbanlibraries.org/filebin/llcc/ 4-7_success_strategy_data_sharing.pdf. Accessed Nov 14, 2019 | Education / Schools | Education / Schools,  Tribal / Local / State / Federal Government- Organized government (bureaucracy) not included in others | 2 | 2 | 2 |  |
| 148 | Justice and Health Connect. The legal landscape of justice and health information sharing. http://www.jhconnect.org/wp-content/uploads/2013/06/ legal-issue-paper-final.pdf. Accessed Dec 19, 2019 | Justice System / Courts | Clinical Health Care, Justice System / Courts | 2 | 2 | 2 |  |
| 149 | Electronic Privacy Information Center (EPIC). The privacy act of 1974. https://epic.org/privacy/1974act/. Accessed Dec 18, 2019 | Organized Government (Tribal/Local/State/Federal) not included in others | Tribal / Local / State / Federal Government- Organized government (bureaucracy) not included in others | 3 | 1 | 3 |  |
| 150 | Substance Abuse and Mental Health Services Administration (SAMHSA). The role of criminal justice data and behavioral health data in whole person care. https://211sandiego.org/wp-content/uploads/2018/04/the-role-of-criminal-justice-data-and-behavioral-health-data-in-whole-person-care.pdf. Accessed Jan 24, 2020 | Mental / Behavioral Health Care | Clinical Health Care,  Criminal Justice - Correctional facilities (jails/prisons),  Mental / Behavioral Health Care,  Public Health (Government) | 2 | 2 | 2 |  |
| 151 | Centers for Medicare & Medicaid Services. Using data to identify housing needs and target resources. https://www.medicaid.gov/state-resource-center/ innovation-accelerator-program/iap-downloads/program-areas/nds-using-data-transcript.pdf. Accessed Nov 18, 2019 | Housing & Homelessness | Clinical Health Care,  Housing & Homelessness,  Mental / Behavioral Health Care,  Social and Human Services | 2 | 3 | 2 |  |
| 152 | All in data. Using electronic health data for community health –part 2. http://www.allindata.org/wp-content/uploads/2018/05/ehr-webinar-part-2-slides_final.pdf. Accessed Sep 25, 2019 | Public Health (Government) | Clinical Health Care,  Health Care Payers,  Public Health (Government) | 2 | 2 | 1 |  |
| 153 | Wisconsin Department of Workforce Development. Vocational rehabilitation: confidentiality. https://dwd.wisconsin.gov/dvr/policy-guidance/ legal/confidentiality.pdf. Accessed Dec 18, 2019 | Social and Human Services | Business,  Social and Human Services | 4 | 3 | 4 |  |
| 154 | Workforce Innovation Technical Assistance Center (WINTAC). WINTAC toolkit –data sharing agreements. http://wintac-s3.s3-us-west-2.amazonaws.com/ topic-areas/datasharingtoolkit_17502_final%28revised%29.pdf. Accessed Nov 14, 2019 | Social and Human Services | Education / Schools, Social and Human Services | 4 | 3 | 4 |  |

**Supplemental Table 2. Laws discussed in fewer than four publicly available resources (n=154)**

| **Discussed in 3 resources** | | |
| --- | --- | --- |
| CAPTA - Child Abuse Prevention and Treatment Act | Children's Internet Protection Act (CIPA), 47 U.S.C. § 254 | Children's Online Privacy Protection Act |
| Confidentiality of Drug Abuse, Alcoholism and Alcohol Abuse, Human Immunodeficiency Virus (HIV) Infection, and Sickle Cell Anemia Medical Records | Confidentiality of Federal Tax returns (26 U.S. Code § 6103) | Copyright Law |
| Cybersecurity Information Sharing Act (CISA) | FDA Regulatory Requirements for Research | Federal Immigration Data Sharing Statutes (e.g., 8 U.S.C. sections 1105, 1373, and 1644) |
| Federal Trade Secrets Act | Homeland Security Act of 2002 | Omnibus Crime Control and Safe Streets Act of 1968 (28 CFR Part 22) |
| Patient Protection and Affordable Care Act | PATRIOT Act | Privacy Protection Act |
| Social Security Act, Title IV-E, Payments for Foster Care and Adoption Services | TANF - Temporary Assistance for Needy Families | Trade Secret Law |
| CAPTA - Child Abuse Prevention and Treatment Act | Children's Internet Protection Act (CIPA), 47 U.S.C. § 254 | Children's Online Privacy Protection Act |
| US Constitution and Amendments |  |  |
| **Discussed in 2 resources** | | |
| Children's Health Insurance Program (CHIP) | Confidentiality Protections Governing Vocational Rehabilitation Records - 34 CFR 361.38 | Fair Credit Reporting Act (FCRA) (1970) |
| Federal Information Security Management Act (FISMA) | Federal Trade Commission Act | Financial Modernization Act (Gramm-Leach-Bliley Act 1999) |
| Fostering Connections to Success and Increasing Adoptions Act | Genetic Information Nondiscrimination Act of 2008 | National Security Act |
| Protection of Pupil Rights Amendment | Social Security Act, Title IV-D, Child Support Enforcement | Social Security Act, Title XVI, Supplemental Security Income for the Aged, Blind and Disabled |
| Social Security Act, Title XX, Block Grants to States for Social Services and Elder Justice | Stored Communications Act |  |
| **Discussed in 1 resource** | | |
| 21st Century Cures Act | Americans with Disabilities Act | Cable Communications Policy Act |
| Child and Family Services Improvement and Innovation Act | Clarifying Lawful Overseas Use of Data (CLOUD) Act | Clayton Act |
| Communications Decency Act of 1996 | Community Services Block Grant Act (42 U.S.C. 9901) | Corporate Governance Annual Disclosure Act (model law) |
| Cybersecurity Enhancement Act of 2015 (6 USC § 151) | Defend Trade Secrets Act | Disclosures by Federal Officials, 18 U.S. Code § 1905 |
| Dodd-Frank Act. | Federal Court Rules | Federal Rules of Evidence and Civil Procedure |
| Food and Drug Administration Amendments Act of 2007 (FDAAA) | Government Performance and Results Act | Higher Education Act |
| Indian Child Welfare Act (ICWA) | Intelligence Reform and Terrorism Prevention Act (IRTPA) of 2004 | Justice System Improvement, 34 U.S.C. Chapter 101 |
| Juvenile Justice and Delinquency Prevention Act | Medicare | Model State Public Health Privacy Act (MSPHPA) |
| Model Vital Statistics and Regulations Act (MVSRA) | National Cybersecurity Protection Act of 2014 | National School Lunch Act (NSLA), 42 U.S.C. §§1751-63 |
| Occupational Safety and Health Act | OMB Circular A-110 | Own Risk Solvency Assessment Act (model law) |
| Patent Law | Pen Register and Trap and Traces Devices Act (Pen/Trap Act) | Public Health Services Act (PHSA) |
| Shelby Amendment | Sherman Act | Social Security Act, Title I |
| Social Security Act, Title III | Social Security Act, Title IX | Social Security Act, Title X |
| Social Security Act, Title XII | Social Security Act, Title XIV | Statewide Automated Child Welfare Information Systems |
| Student Digital Rights and Privacy Act | Supplemental Nutrition Program for Women, Infants and Children (WIC) (7 CFR §246.26) | The VA Claims Confidentiality Statute |
| Title II of the Trade Act of 1974, as amended | Title V of the Older Americans Act of 1965 | Toxic Substances Control Act (TSCA) |
| Uninterrupted Scholars Act | Violence Against Women Act | Wiretap Act |

**NOTE:** Names of laws are as described in the resources. While all of the above laws were discussed in relation to data sharing issues, we did not independently verify whether all the above cited laws in fact have legal data protection or sharing provisions.

**Supplemental Table 3. Data type discussed in the resources (n=154)**

| **Data type** | **In number of resources** |
| --- | --- |
| Service (EHRs, case management, education records) - Encounters, provision of services | 95 |
| Administrative - Payment, eligibility, claims, enrollment | 93 |
| Clinical Health | 62 |
| Surveillance - Includes epi and Behavioral Risk Factor Surveillance System | 19 |
| Data (not specified) | 16 |
| Commercial Consumer - Generated by private business; consumer purchase/preference | 12 |
| Not expressly discussed in resource | 5 |
| Personal Demographic - Descriptive | 5 |
| Research-generated surveys and assessments | 4 |
| Geographic - Descriptive data about the attributes of a specified area, i.e., zip codes, GIS databases | 2 |
